# SssP1, a Fimbria-like component of *Streptococcus suis*, binds to the vimentin of host cells and contributes to bacterial meningitis

**DOI:** 10.1371/journal.ppat.1010710

**Published:** 2022-07-19

**Authors:** Zihao Pan, Peijuan He, Yue Zhang, Qibing Gu, Shengsheng Chen, Yong Yu, Jing Shao, Kaicheng Wang, Zongfu Wu, Huochun Yao, Jiale Ma

**Affiliations:** 1 College of Veterinary Medicine, Nanjing Agricultural University, Nanjing, China; 2 OIE Reference Laboratory for Swine Streptococcosis, Nanjing, China; 3 MOE Joint International Research Laboratory of Animal Health and Food Safety, Nanjing Agricultural University, Nanjing China; 4 Shanxi Animal Health and Slaughtering Management Station, Xian, China; 5 College of Veterinary Medicine, Henan Agricultural University, Zhengzhou, China; 6 Sichuan Animal Disease Prevention and Control Center, Chengdu, China; 7 Key Laboratory of Animal Biosafety Risk Prevention and Control (South), China Animal Health and Epidemiology Center, Qingdao, China; Lunds universitet Medicinska fakulteten, SWEDEN

## Abstract

*Streptococcus suis* (*S*. *suis*) is one of the important pathogens that cause bacterial meningitis in pigs and humans. Evading host immune defences and penetrating the blood-brain barrier (BBB) are the preconditions for *S*. *suis* to cause meningitis, while the underlying mechanisms during these pathogenic processes are not fully understood. By detecting the red blood and white blood cells counts, IL-8 expression, and the pathological injury of brain in a mouse infection model, a serine-rich repeat (SRR) glycoprotein, designated as SssP1, was identified as a critical facilitator in the process of causing meningitis in this study. SssP1 was exported to assemble a fimbria-like component, thus contributed to the bacterial adhesion to and invasion into human brain microvascular endothelial cells (HBMECs), and activates the host inflammatory response during meningitis but is not involved in the actin cytoskeleton rearrangement and the disruption of tight junctions. Furthermore, the deletion of *sssP1* significantly attenuates the ability of *S*. *suis* to traverse the BBB *in vivo* and *in vitro*. A pull-down analysis identified vimentin as the potential receptors of SssP1 during meningitis and following Far-Western blot results confirmed this ligand-receptor binding mediated by the NR2 (the second nonrepeat region) region of SssP1. The co-localisation of vimentin and *S*. *suis* observed by laser scanning confocal microscopy with multiplex fluorescence indicated that vimentin significantly enhances the interaction between SssP1 and BBB. Further study identified that the NR_216-781_ and NR_1711-2214_ fragments of SssP1 play critical roles to bind to the BBB depending on the sialylation of vimentin, and this binding is significantly attenuated when the antiserum of NR_216-781_ or NR_1711-2214_ blocked the bacterial cells, or the vimentin antibody blocked the BBB. Similar binding attenuations are observed when the bacterial cells were preincubated with the vimentin, or the BBB was preincubated with the recombinant protein NR_216-781_, NR_1711-2214_ or sialidase. In conclusion, these results reveal a novel receptor-ligand interaction that enhances adhesion to and penetration of the BBB to cause bacterial meningitis in the *S*. *suis* infection and highlight the importance of vimentin in host-pathogen interactions.

## Author summary

*Streptococcus suis* (*S*. *suis*) is considered an important zoonotic pathogen capable of causing meningitis in humans. Penetrating the blood-brain barrier (BBB) is one of the preconditions for *S*. *suis* to cause meningitis, while its underlying mechanism is incompletely understood. Here we identified a previously uncharacterised pathogenic mechanism associated with *S*. *suis* meningitis mediated by the interaction between bacterial SRR glycoproteins and a host cytoskeletal component. During the bacterial infection, SRR protein SssP1 is exported to assemble a fimbria-like component, which drives a strong binding effect with the BBB depending on the sialylation of vimentin. This interaction contributes to the bacterial adhesion to and penetration of the BBB and induces a robust inflammatory response during meningitis. This overall observation underscores the significance of host cell surface vimentin interactions in microbial pathogenesis and markedly improves our understanding of host barrier penetration during meningitis.

## Introduction

*Streptococcus suis* (*S*. *suis*) is considered an emerging zoonotic pathogen, causing severe infections in pigs and humans, in which meningitis is one of the most life-threatening manifestations [1]. To cause meningitis, *S*. *suis* must contact and traverse the blood-brain barrier (BBB), separating the circulating blood from the brain extracellular fluid in the central nervous system [2]. As an essential defensive structure, the BBB mainly comprises brain microvascular endothelial cells, neuroglial cells, and peripheral cells [3, 4]. The processes of bacterial pathogens penetrating the BBB are multifactorial and require complicated interactions with host cells [5, 6]. Different pathogens rely on varied mechanisms to penetrate this barrier via transcellular or paracellular routes or through infected Trojan horse mechanisms [7].

Numerous studies focus on meningitis caused by *S*. *suis* and try to clarify the underlying mechanisms tentatively. Different biological techniques were employed to screen the virulence factors required for *S*. *suis* meningitis infection, including the selective capture of transcribed sequences (SCOTS) approach on a porcine brain microvascular endothelial cell infection model [8], the bacterial transcriptomic analysis after co-incubation with the swine cerebrospinal fluid (CFS) *in vitro* [9], and the TnYLB-1 transposon mutagenesis strategy using a BBB infection model *in vitro* [10]. Although many vital genes, small RNAs (sRNA) and extracellular components were successfully identified using these techniques, only a few ones were verified to facilitate meningitis during *S*. *suis* infection experimentally, such as the sRNA rss04 regulating CPS synthesis [11], the Serine/Threonine protein kinase destroying the tight junctions (TJs) protein claudin-5 of human brain microvascular endothelial cells (HBMECs) [12], the MRP inhibiting the adherens junctions (AJs) protein p120-catenin of BBB [13], and the suilysin inducing actin rearrangement of BBB [14]. These findings revealed the processes of transcellular and paracellular traversal during *S*. *suis* crossing the BBB models *in vitro*, while the underlying pathogenesis of meningitis caused by *S*. *suis in vivo* is not yet understood fully since the lack of high-virulent strains and suitable animal infection models to reproduce the typical meningitis symptoms perfectly.

In previous studies, we identified the *S*. *suis* strain CZ130302 that could cause acute meningitis with the representative symptoms of central nervous system infection (by video records) in the mouse infection model [15]. We then confirmed this phenotype that significantly correlated with a 4,647 aa Serine-rich repeat (SRR) glycoprotein, which transported by the SecY2/A2 (also known as accessory Sec) system [16]. Numerous studies have demonstrated that the SRR family proteins played critical roles in bacterial pathogenicity by mediating the attachment to host cell surfaces using the nonrepeat (NR) domains [17,18]. In *Streptococcus pneumoniae*, the NR domain of PsrP binds to the lung epithelial cell surface factor keratin 10, thus significantly promoting the pneumococcal infection [19]. While the NR domains of Hsa and GspB were confirmed to enable *Streptococcus gordonii* to effectively colonise the damaged endocardium and cause infective endocarditis via the attachment to the sialylated ligands on the platelet membrane [20].

Vimentin is broadly expressed in mesenchymal cells of mesodermal origin, such as platelets, neutrophils, activated macrophages, vascular endothelial cells, and brain microvascular endothelial cells [21–26], and historically is viewed as a cytosolic intermediate filament protein that forms static cytoskeletal networks important for cell structural integrity [27,28]. Interestingly, vimentin also involves in many cellular processes such as cell adhesion, cellular signalling, autophagy, and bacterial infections [29–32]. Several studies have shown that vimentin can activate the inflammation pathways by regulating the activity of NOD2 and NLRP3 inflammasome [33–35]. The surface proteins of bacterial pathogens play critical roles in causing meningitis via the interaction with the host components (such as the vimentin) [36]. For example, the BspC of Group B Streptococcal interacts with vimentin to promote adherence to brain endothelium to cause meningitis [37]. The surface-localised vimentin can mediate the adhesion of *Listeria monocytogenes* and *Escherichia coli* to HBMECs via their virulence factor InlF and IbeA, respectively [27,38].

In this study, our group identified a previously uncharacterised pathogenic mechanism associated with meningitis mediated by the interaction between a bacterial SRR glycoprotein and a host cytoskeletal component. During the bacteremia infection by *S*. *suis*, SRR protein SssP1 is exported to assemble a fimbria-like component, which drives a strong binding effect with the vimentin of BBB. This interaction contributes to the bacterial adhesion to and penetration of the BBB and induces a robust inflammatory response during meningitis.

## Results

### SssP1 is a crucial facilitator in the process of causing meningitis

The *S*. *suis* strain CZ130302, isolated from piglet meningitis case, was confirmed to cause typical meningitis symptoms in mouse infection model [15], thus was used to explore the potential mechanism of bacterial meningitis in this study. The SssP1, a 4,647 aa SRR glycoprotein (AWD32147.1) of CZ130302, was identified to assemble a fimbria-like component [16], and significantly associated with the occurrence of bacterial meningitis. Therefore, a *sssP1* deletion mutant was constructed. The transcriptional levels of more than 50 upstream and downstream genes were assessed without significant changes *in vivo* and *in vitro*, which partially verified that the deletion operation did not cause significant polarity effects. It is regrettable for the inoperability of constructing the complementary strain by reintroducing a 13,944 bp *sssP1* gene. Therefore, a mutant (SssP1^T182A^) with a stop codon at position 182 bp was constructed by artificially changing T to A, and identified to display similar phenotypes with the Δ*sssP1* strain in growth, pathogenicity and deficiency of fimbria-like structure [16], which indicated that Δ*sssP1* is a non-polar deletion mutant.

**Fig 1:**
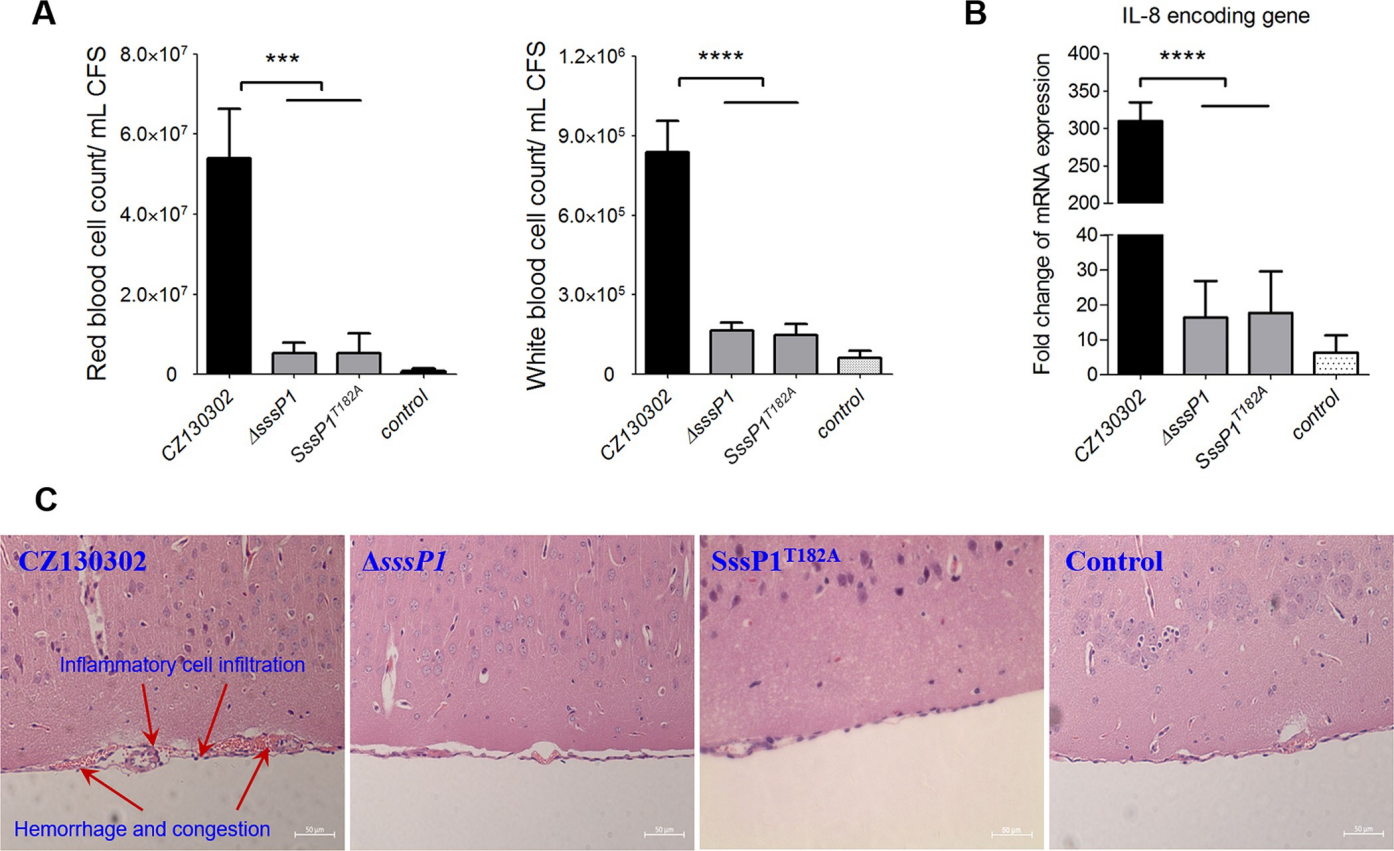
SssP1 is critical for *S*. *suis* to cause meningitis. Since the inoperability of constructing the complementary strain by reintroducing a 13,944 bp *sssP1* gene, a mutant (SssP1^T182A^) with a stop codon at position 182 bp was constructed by artificially changing T to A, which was used as an alternative method to confirm that Δ*sssP1* is a non-polar deletion mutant. (A) The red blood and white blood cell counts of cerebrospinal fluid (CFS) from the mice infected with the indicated strains. Data are represented as mean ± SEM of three independent repeats (**** *P* < 0.0001, *** *P* < 0.001). (B) Transcriptional level of IL-8 encoding gene in the brains of the mice infected with the indicated strains. Data are represented as mean ± SEM of three independent repeats (**** *P* < 0.0001). (C) Histopathological analysis of the brains from the mice challenged with CZ130302, Δ*sssP1* or SssP1^T182A^. Scale, 50 μm.

A mouse infection model challenged by tail intravenous injection was used to assess the potential roles of SssP1 in meningitis, and the cerebrospinal fluids (CFS) were collected at 12 h post-infection. As shown in Fig 1A, both the red blood cell count and white blood cell count from the Δ*sssP1* and SssP1^T182A^ infection group were significantly reduced to a comparable level of blank control compared with the wild-type infection group. The activation of inflammatory response is a common feature in bacterial meningitis, and interleukin-8 (IL-8) is a general marker for inflammation level assessment. In this study, the brain tissues of mice infected with Δ*sssP1* and SssP1^T182A^ showed a significantly lower transcriptional level of IL-8 than wild-type at 12 h post-infection (Fig 1B) (*P* < 0.0001), suggesting that the gene *sssP1* is significantly associated with inflammation. Furthermore, there were marked lesions in mice brains challenged with CZ130302 strain, like bleeding point, cell swelling, brain vascular dilatation and congestion, and inflammatory cell infiltration, as shown under the microscope (Fig 1C). However, the group infected with Δ*sssP1*, SssP1^T182A^ or PBS has no obviously histopathological changes. Those results suggested that the gene *sssP1* facilitates the process of *S*. *suis* causing meningitis.

### Deletion of *sssP1* significantly attenuates the ability of *S*. *suis* to penetrate the BBB *in vivo*

**Fig 2:**
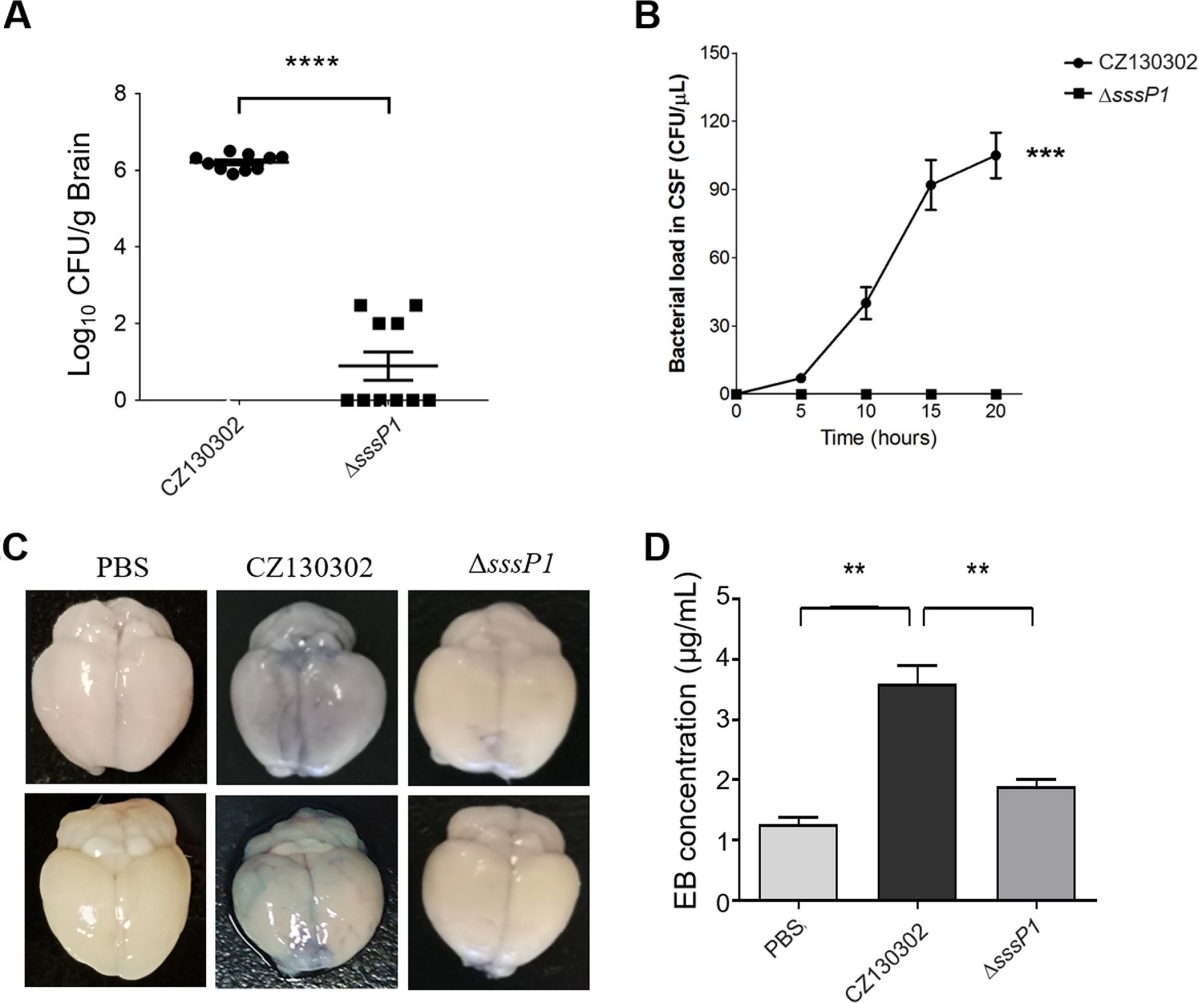
SssP1 is required for *S*. *suis* to cross the BBB *in vivo*. (A) The bacterial load of the wild-type and Δ*sssP1* strains in the brain of the mice infected with the indicated strains. Data are represented as mean ± SEM of triplicate samples (**** *P* < 0.0001). (B) The curves showed the bacterial loads in CFS of the mice challenged with different *S*. *suis* strains until 20 h post-infection. Mean values and SEMs of triplicate samples are indicated. (C, D) Evans Blue (EB) permeability in brains of the mice infected with the indicated strains. EB concentration of the infected brains was measured. Data are represented as mean ± SEM of triplicate samples (** *P* < 0.01).

The bacterial loads of CZ130302 and Δ*sssP1* in CFS and brain tissue weremeasured after tail intravenous injection in mouse infection model to evaluate the effect of the *sssP1* gene on the capacity of invasion and colonisation during bacterial meningitis. As shown in Fig 2A, the bacterial load of Δ*sssP1* strain in brain tissue was also notably reduced in comparison to wild-type strain at 20 h post-infection (*P* < 0.0001). The bacterial load of CZ130302 in CFS increased gradually to approximately 100 CFU/μL at 20 h post-infection, while CFS from the mice challenged with Δ*sssP1* strain, there were no bacterial cells detected (Fig 2B). These results suggested that SssP1 might promote *S*. *suis* to penetrate the BBB *in vivo*. Thus, the Evans Blue (EB) dye permeability assay was performed to assess the integrity of the BBB in mice challenged with the indicated *S*. *suis* strains. Accordingly, the brains of mice infected with Δ*sssP1* showed lower EB than the wild-type strain (Fig 2C and 2D) (*P* < 0.01). These observations suggest that the deletion of *sssP1* significantly attenuates the ability of *S*. *suis* to penetrate the BBB in the mouse infection model.

### Deletion of *sssP1* significantly decreases the destruction of BBB models established by different cells *in vitro*

**Fig 3:**
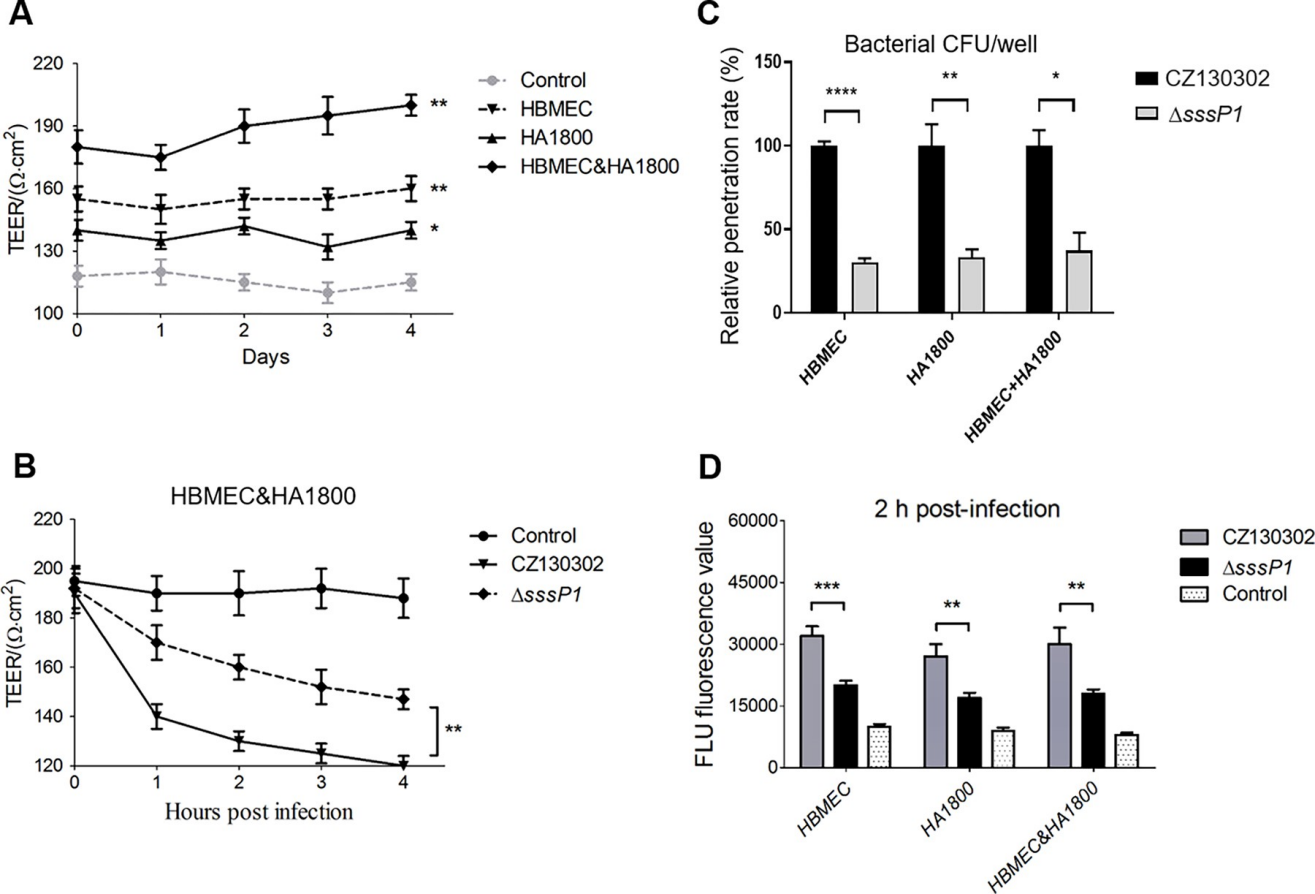
SssP1 promotes *S*. *suis* to cross the BBB *in vitro*. All data are represented as mean ± SEM of triplicate samples (**** *P* < 0.0001, ** *P* < 0.01, *P < 0.05). (A) The curves showed the TEER values of three types of models within 4 days. The HBMECs and astrocyte cell HA1800 were respectively or jointly inoculated into a transwell chamber to establish three types of BBB models *in vitro* (S1 Fig). (B) The curves showed the TEER values of the HBMEC&HA18000 joint BBB model infected with the indicated *S*. *suis* strains. (C) The penetration rates of three types of models infected with the indicated strains were measured, and the data showed the calculated value of the bacterial CFU in the bottom chamber/ upper + bottom chambers. (D) The fluorescence values of the bottom chambers at OD428 were measured to show the permeability of Fluorescein (FLU) in three type of BBB models infected with the indicated bacterial strains. The boiled CZ130302 was used as control.

The establishment of the BBB model *in vitro* provides a stable qualitative and quantitative evaluation tool for further studying the mechanism of bacterial meningitis [39, 40]. In this study, the human brain microvascular endothelial cell (HBMEC) and astrocyte cell HA1800 were respectively or jointly inoculated into a Transwell chamber to establish three types of BBB models *in vitro* (S1 Fig). All three models showed significantly higher trans-endothelial electrical resistance (TEER) values than the blank control (Fig 3A), indicating that the barrier had already formed successfully. Significantly, the HBMEC& HA1800 model showed the highest TEER value. Thus, it was used to assess the potential roles of SssP1 in the destruction of BBB by monitoring the changing curve of TEER values. Expectedly, the deletion of *sssP1* caused a significantly less decrease of TEER value compared to the wild type in the inoculated BBB model (Fig 3B). We then measured the bacterial load within the bottom chambers, which showed the ability of *S*. *suis* crossing the BBB *in vitro*. In 2 h post-infection, wild-type strain showed significantly higher penetrability than Δ*sssP1* in all Transwell chamber BBB models (Fig 3C) (*P* < 0.05; *P* < 0.01; *P* < 0.0001), suggesting that SssP1 affects the ability of bacteria to penetrate the BBB *in vitro*. In order to detect the BBB integrity more sensitively, the sodium Fluorescein (FLU) was added to this infection model and measured the fluorescence value of the bottom chambers at OD428 to show the destructiveness of the indicated bacterial strains to the BBB. As shown in Fig 3D, the solutions showed significantly lower FLU values treated by Δ*sssP1* than wild-type strain at 2 h post-infection. Similar results were observed in the human umbilical vein endothelial cell (HUVEC) model after bacterial infection (S2 Fig). This result indicated that the penetration of Δ*sssP1* to BBB was significantly attenuated compared to the wild-type strain.

### SssP1 contributes to bacterial adhesion and invasion, and activates the host inflammatory response during meningitis

**Fig 4:**
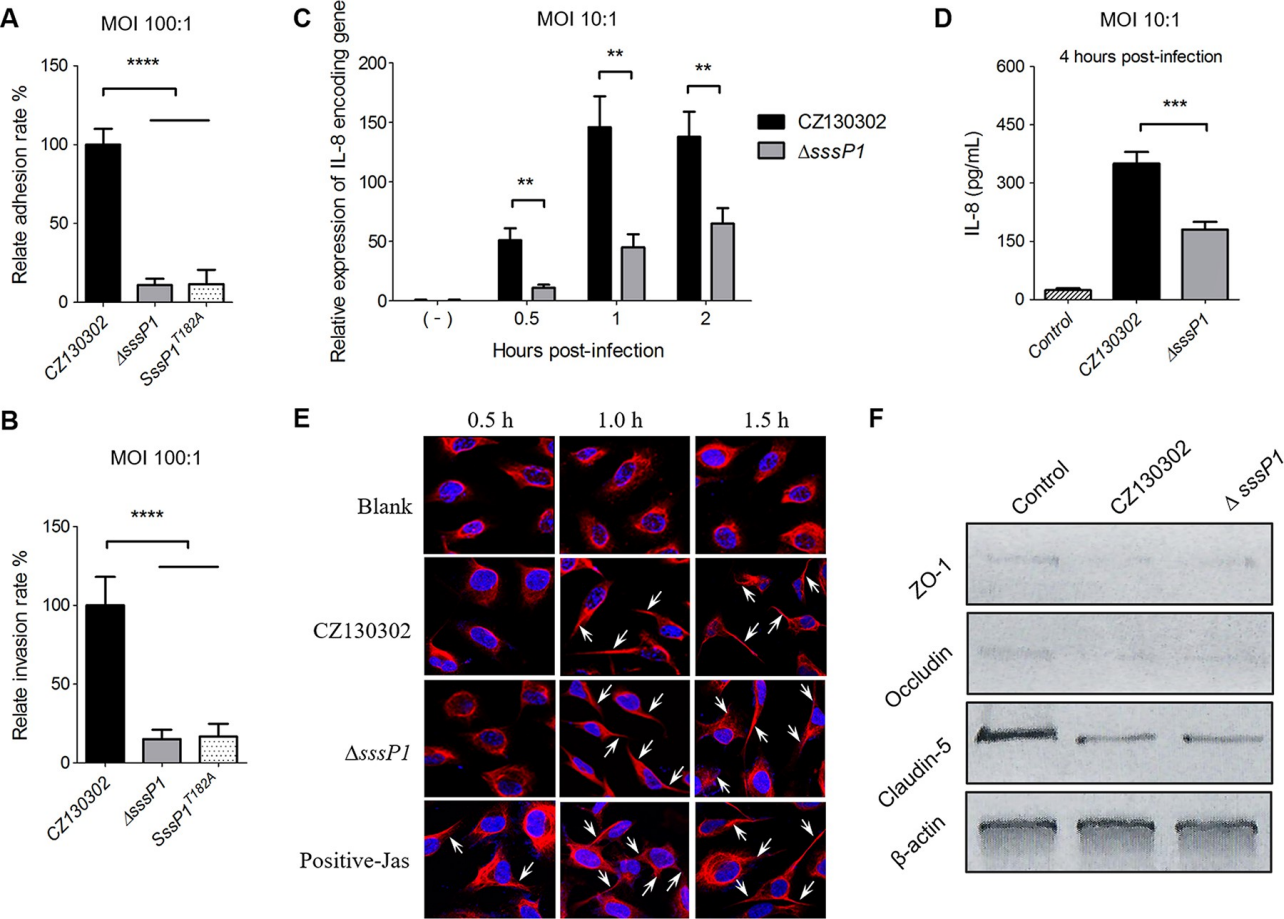
The *sssP1* plays significant roles in adherence and invasion. All data are represented as mean ± SEM of triplicate samples (**** *P* < 0.0001, *** *P* < 0.001, ** *P* < 0.01). (A, B) The adhesion and invasion levels of the indicated *S*. *suis* strains to HBMECs were detected at the MOI 100:1. (C) The transcriptional level of IL-8 encoding gene in the HBMECs interacted with different *S*. *suis* strains were detected using an RT-qPCR assay at the indicated times post-infection. (D) The releasing levels of IL-8 in the cultural supernatant of the HBMECs infected with different *S*. *suis* strains were measured at 4 h post-infection. (E) Cytoskeletal changes of HBMECs in response to infection by *S*. *suis* CZ130302 and Δ*sssP1*. Cells were stained with phalloidin to show actin filaments and were imaged using confocal microscopy. Control HBMECs were incubated without bacteria. Otherwise, HBMECs were incubated with *S*. *suis* for 0.5, 1.0, and 1.5 h before phalloidin staining. (F) Levels of the three major components of TJs, ZO-1, occludin and claudin-5, were detected using a Western blot assay to the total protein samples from the HBMECs infected with the indicated strains. β-actin was used as a loading control.

The following assays were performed to explore the underlying mechanisms of SssP1 in the bacterial penetration of BBB during *S*. *suis* infection. As shown in Fig 4A, the adhesion rates of Δ*sssP1* and SssP1^T182A^ to HBMECs were decreased by nearly 90% compared with the wild-type strain (*P* < 0.0001), indicating that SssP1 has a strongly adhesive ability to HBMECs, which may enhance the virulence to cause meningitis. Likewise, the abilities of Δ*sssP1* and SssP1^T182A^ invading the HBMECs were significantly attenuated compared with the wild-type strain (Fig 4B) (*P* < 0.0001). The invasion/ahersion ratios were then measured, and the values stayed around 0.18 both in the CZ130302 and Δ*sssP1* infection groups, suggesting that the attenuation of bacterial invasion in Δ*sssP1* might be caused by the adhesion defect indirectly.

The higher bacterial loads of wild-type strain in brain tissue might induce higher IL-8 levels (Figs 1B and 2A). Thus, the defect of Δ*sssP1* inducing inflammatory response needs confirmation whether it was caused by indirectly reducing bacterial load or by the interaction of SssP1 with the host directly. We then measured the related data in HBMECs after interacting with the equal bacterial cells of Δ*sssP1* or wild-type strains at the MOI 10:1. The results showed that the transcriptional level of IL-8 encoding gene in HBMECs interacted with Δ*sssP1* after 0.5 h were significantly lower than the wild-type strain (Fig 4C) (*P* < 0.01). Furthermore, the releasing levels of IL-8 at 4 h post-infection also showed a significant decrease infected by the Δ*sssP1* compared with the wild-type strain (Fig 4D) (*P* < 0.001). These observations suggested that SssP1 can directly stimulate HBMECs to produce a more robust inflammatory response.

### SssP1 is not involved in the actin cytoskeleton rearrangement and disruption of TJs during *S*. *suis* infection

Many meningitis-causing bacterial pathogens cross the BBB by modifying or rearranging the actin cytoskeleton and disrupting the epithelial tight junction proteins (TJs) [41,42], including *S*. *suis* [43]. To determine whether SssP1 affects actin filaments, HBMECs were infected with CZ130302 or Δ*sssP1* at the MOI 10:1 and then stained with Alexa Fluor 660 phalloidin. Cells were serum-starved to inactivate Rho family proteins and enhance the response to activators [44]. In the absence of *S*. *suis*, there is a notable paucity of actin filaments visible in HBMECs at the three time points (Fig 4E). In the presence of *S*. *suis*, both Δ*sssP1* and wild-type strains caused the production of stress fibres, filopodia, lamellipodia, and a long protrusion of actin filament stained in HBMECs after 1 h post-infection (Fig 4E). However, it was clear that the distribution of actin filaments showed no apparent differences in the presence of CZ130302 and Δ*sssP1* at the same time. Otherwise, ZO-1, occludin, and claudin-5 are significant components of TJs [45,46], and these proteins are localised at TJs of the BBB endothelial cells. Western blotting showed that *S*. *suis* caused the significant decreases of these three proteins on the infected monolayer of HBMECs at 8 h post-infection (Fig 4F), while there were no significant differences observed between the Δ*sssP1* and wild-type strain treated cells. These data suggested that both the rearrangement of the actin cytoskeleton and disruption of TJs caused by the *S*. *suis* infection is independent of SssP1.

### Screening of the potential receptors of HBMECs which amplify the pathogenic effects of SssP1

**Fig 5:**
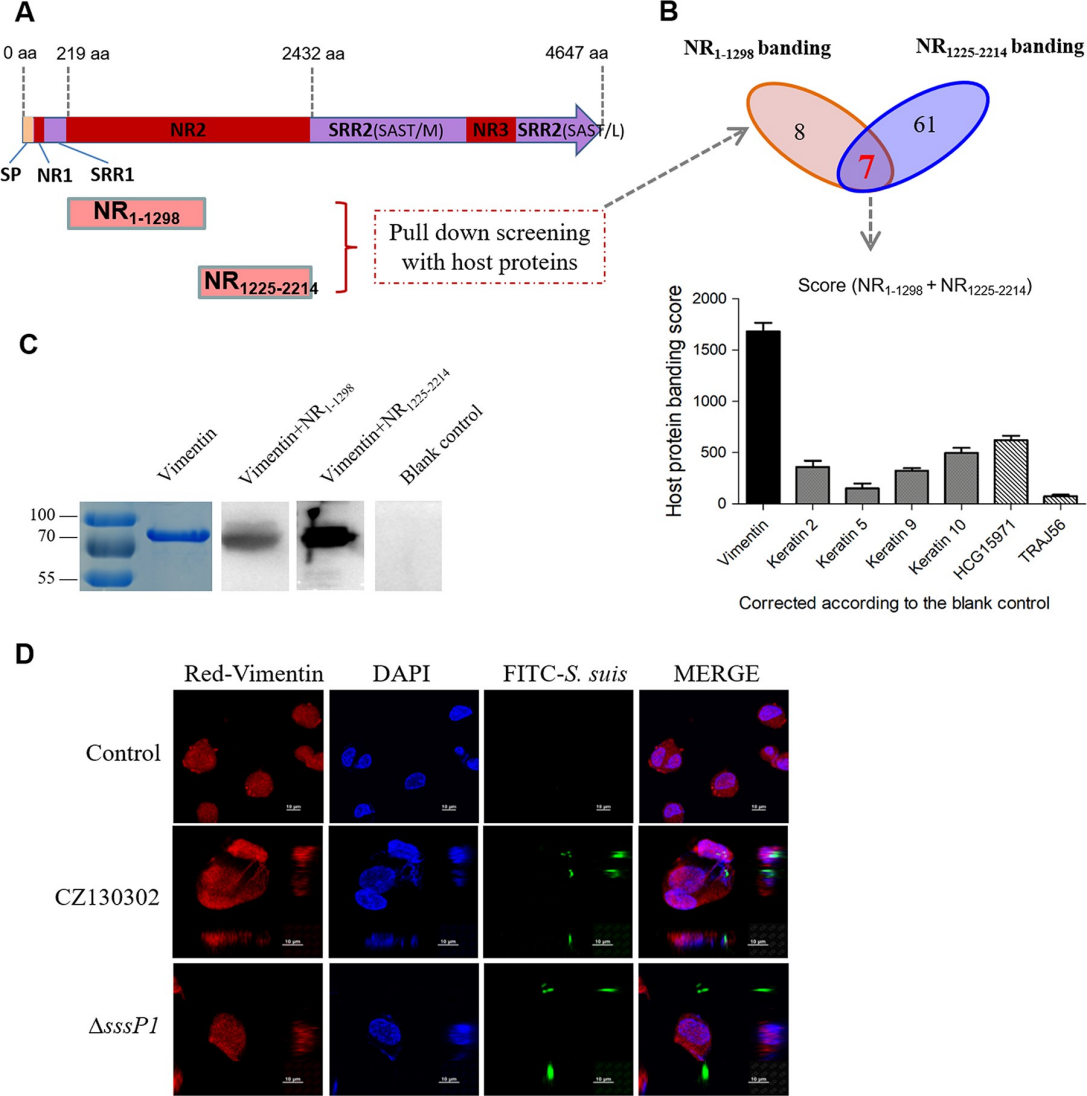
Vimentin of HBMECs was screened as a vital receptor of SssP1. (A) Schematic diagram of the domain architecture of SssP1 and the location of NR_1-1298_ and NR_1225-2214_. (B) The analysis of the screened host proteins by pull-down assays of SssP1-NR_1-1298_ and SssP1-NR_1225-2214_ is based on the data shown in Tables 1 and S2. The binding score represents the ability to interact with SssP1 based on the protein loads measured in the pull-down assays. (C) The Far-Western blot was performed to identify the interaction between vimentin and the indicated NR fragments of SssP1. (D) Visual observation of the localisation of vimentin and *S*. *suis* co-incubated with the HBMECs. The vimentin skeleton was stained by indirect immunofluorescence (IIF) in red, the nuclei were counterstained with DAPI in blue, and the *S*. *suis* cells with green fluorescent plasmid pKSM410-rss29 were observed in green.

Normally, most SRRPs contain two nonrepeat regions (i.e., NR1 and NR2) and two serine-rich repeat regions (i.e., SRR1 and SRR2) [47]. Like other members of the SRRPs family [48], the basic structure of SssP1 is conserved. However, the ligand-binding region (NR2 domain) between SRR1 and SRR2 is distinctive among SRRPs from different species and strains [47,49]. It is why different SRRPs have different interacting receptors in the pathogenic process. Therefore, to find the target receptors on the brain microvascular endothelial cells which amplify the pathogenic effects of SssP1 in adhesion, invasion, and inflammatory response, the NR2 domain of SssP1 was divided into two segments (NR_1-1298_ and NR_1225-2214_) to express by prokaryotes since the large and complication of its structure (Fig 5A). Then, the two segments were purified and interacted with HBMECs for the pull-down screening, respectively. The results showed that 15 and 69 candidate proteins targeted by NR_1-1298_ and NR_1225-2214_ were identified, respectively (S2 Table). Since the blank control group also detected eight proteins, although they showed lower interaction scores, we corrected the data according to the blank control and listed the proteins showing top 7 scores as the potential candidates from NR_1-1298_ and NR_1225-2214_ binding groups in Table 1, respectively.

**Table 1:** The potential receptor proteins of HBMEC were screened jointly by SssP1-NR_1-1298_ and SssP1-NR_1225-2214_ binding.

Accession	Score*	Mass	emPAI	Protein description
**Screening of receptor proteins interacting with protein SssP1-NR**_**1-1298**_				
sp|P35527|K1C9_HUMAN	181	62255	0.76	Keratin, type I cytoskeletal 9 OS = Homo sapiens OX = 9606 GN = KRT9
sp|P13645|K1C10_HUMAN	113	59020	0.92	Keratin, type I cytoskeletal 10 OS = Homo sapiens OX = 9606 GN = KRT10
sp|P35908|K22E_HUMAN	107	65678	0.41	Keratin, type II cytoskeletal 2 epidermal OS = Homo sapiens OX = 9606 GN = KRT2
**sp|P08670|VIME_HUMAN**	**101**	**53676**	**0.27**	**Vimentin OS = Homo sapiens OX = 9606 GN = VIM**
sp|P13647|K2C5_HUMAN	77	62568	0.23	Keratin, type II cytoskeletal 5 OS = Homo sapiens OX = 9606 GN = KRT5
Q1KLZ0_HUMAN	71	42052	0.25	HCG15971, isoform CRA_a OS = Homo sapiens OX = 9606 GN = PS1TP5BP1
A0A075B6Z2_HUMAN	15	2220	1.81	T cell receptor alpha joining 56 (Fragment) OS = Homo sapiens OX = 9606 GN = TRAJ56
**Screening of receptor proteins interacting with protein SssP1-NR**_**1225-2214**_				
**sp|P08670|VIME_HUMAN**	**1583**	**53676**	**2.37**	**Vimentin OS = Homo sapiens OX = 9606 GN = VIM**
Q1KLZ0_HUMAN	555	42052	2.62	HCG15971, isoform CRA_a OS = Homo sapiens OX = 9606 GN = PS1TP5BP1
sp|P13645|K1C10_HUMAN	385	59020	2.12	Keratin, type I cytoskeletal 10 OS = Homo sapiens OX = 9606 GN = KRT10
sp|P35908|K22E_HUMAN	254	65678	1.08	Keratin, type II cytoskeletal 2 epidermal OS = Homo sapiens OX = 9606 GN = KRT2
sp|P35527|K1C9_HUMAN	142	62255	0.59	Keratin, type I cytoskeletal 9 OS = Homo sapiens OX = 9606 GN = KRT9
sp|P13647|K2C5_HUMAN	78	62568	0.29	Keratin, type II cytoskeletal 5 OS = Homo sapiens OX = 9606 GN = KRT5
A0A075B6Z2_HUMAN	63	2220	1.81	T cell receptor alpha joining 56 (Fragment) OS = Homo sapiens OX = 9606 GN = TRAJ56

*The binding score was corrected according to the blank control.

### Vimentin mediates the interaction between SssP1 and HBMECs

Further analysis showed that seven candidate proteins have the potential to interact with the NR_1-1298_ and NR_1225-2214_ jointly (Fig 5B), and the protein named vimentin showed the highest score, which represents the ability to interact with SssP1 based on the protein loads measured in the pull-down assays. Far-Western blot assay confirmed that the NR_1-1298_ and NR_1225-2214_ could directly interact with vimentin (Fig 5C), while the other two selected proteins, keratin 2 and 10, showed negative results. The green fluorescent tracer plasmid pKSM410-rss29 was constructed and introduced into a wild-type strain and Δ*sssP1* for the following fluorescence microscope, respectively (S3 Fig), to visualise the localisation of *S*. *suis* and vimentin in brain endothelial cells. HBMECs infected with either CZ130302 or Δ*sssP1* were fixed but not permeabilised to permit labelling of only extracellular bacteria and surface-expressed vimentin by incubating with antibodies to vimentin. We observed that the surface vimentin co-localised with the bacterial cells of the wild-type strain, while this was not observed for HBMECs infected with the Δ*sssP1* mutant (Fig 5D). These results suggested that SssP1 is required for the interaction with host extracellular vimentin during *S*. *suis* infection.

### NR_216-781_ and NR_1711-2214_ of SssP1 play critical roles to bind to vimentin of HBMECs

**Fig 6:**
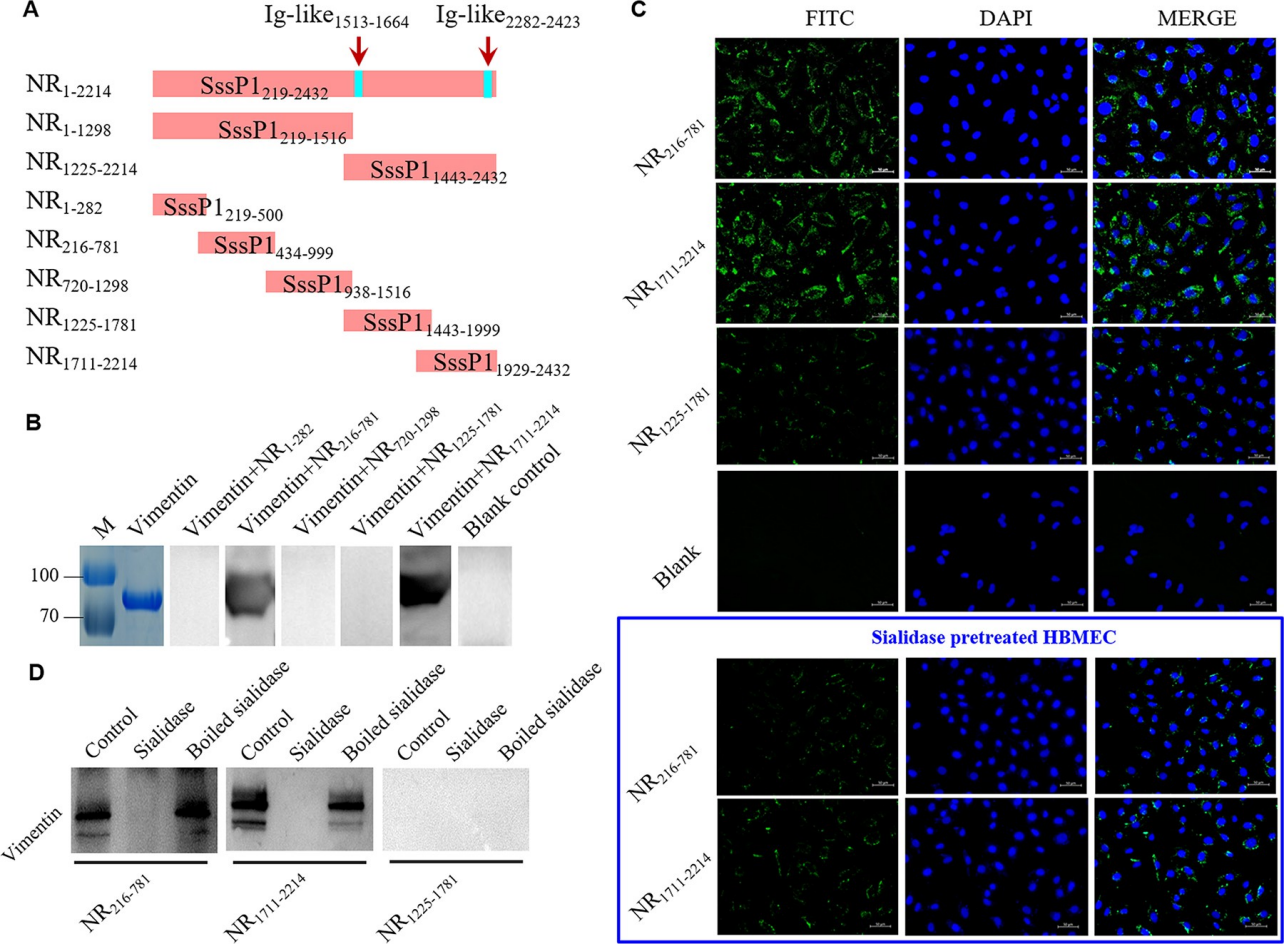
Identification of the smaller fragments required for SssP1_-NR2_ attachment to vimentin. (A) Schematic diagram of the NR domain divided into 5 fragments according to the protein structure. (B) The Far-Western blot was performed to identify the interaction between vimentin and the indicated fragments of NR domain. (C) Contributions of the NR_216-781_ and NR_1711-2214_ fragments in bacterial adhesion to HBMECs. Several indicated cell samples were pretreated for 0.5 h at 37°C using the sialidase (Sigma). The images show the DAPI-stained nuclei in blue, and the adhered proteins detected by goat anti-mouse IgG-FITC in green. The NR_1225-1781_ was used as control. (D) The Far-Western blot analyses of NR domains using the sialidase (Sigma) pretreated vimentin (for 0.5 h at 37°C). The hyaluronidase (Sigma) pretreated vimentin was used as control.

To determine the more accurate/smaller fragment that is required to mediate SssP1 attachment to vimentin, the NR2 domain was re-divided into five overlapping recombination fragments (NR_1-282_, NR_216-781_, NR_720-1298_, NR_1225-1781_ and NR _1711–2214_) according to its structure and function (Fig 6A), and immunising rabbits prepared the polyclonal antibodies against them, respectively. Far-Western blot results verified that two of the five smaller recombinant proteins, NR_216-781_ and NR_1711-2214_, could directly interact with vimentin (Fig 6B), while the remaining three samples and the blank control could not detect the specific bands. In addition, both the proteins of NR_216-781_ and NR_1711-2214_ showed an adhesive ability to HBMECs detected by indirect immunofluorescence (Fig 6C), and the NR_1225-1781_ and blank control showed the negative result correctly. These results demonstrate that the NR domains NR_216-781_ and NR_1711-2214_ of SssP1 can promote the interaction with HBMECs via vimentin to activate its following pathogenic roles during *S*. *suis* infection.

### The sialylation of vimentin is required for the SssP1 binding

Many SRR proteins from diverse pathogens have been reported to utilise their siglec-like (Ig-like) domains to bind platelet sialoglycans preferentially, such as SrpA, GspB, and Hsa [20]. We then used the Web service of CD search and SWISS MODEL to analyze the potential domains within SssP1-NR region, and found that NR_1711-2214_ contains an Ig-like domain and a herpes virus major outer envelope glycoprotein (BLLF1) domain, while NR_216-781_ does not contain any known domains (Fig 6A). Otherwise, the NR_1225-1781_ contains another Ig-like domain, while it could not bind to the vimentin (Fig 6B and 6C). Therefore, whether the cell surface vimentin contains sialic acid modifications and whether the sialic acids are necessary for the SssP1-vimentin interaction need to be addressed. The sialidase (Sigma) was used to pretreat the vimentin protein and HBMECs. The Far-Western blot assay showed that sialidase treatment abolished the NR_216-781_ and NR_1711-2214_ binding to vimentin (Fig 6D), while the hyaluronidase (Sigma) and bioled sialidase treatment had no effect on this phenotype. Significantly different from the original cells, the HBMECs pretreated by sialidase significantly reduced the NR_216-781_ and NR_1711-2214_ bindings (Fig 6C), further suggesting that both the above fragments interacted with vimentin in a sialylation-dependent manner. Furthermore, the sialidase treatment caused the bacterial adhesion of strain CZ130302 to HBMECs reduced to the similar level of Δ*sssP1* (S4 Fig), suggesting that the sialylation of cell surface was required for the SssP1 mediated bacterial adhesion.

### The blocking of vimentin, NR_216-781_ or NR_1711-2214_ attenuates the ability of *S*. *suis* adhering to HBMECs

**Fig 7:**
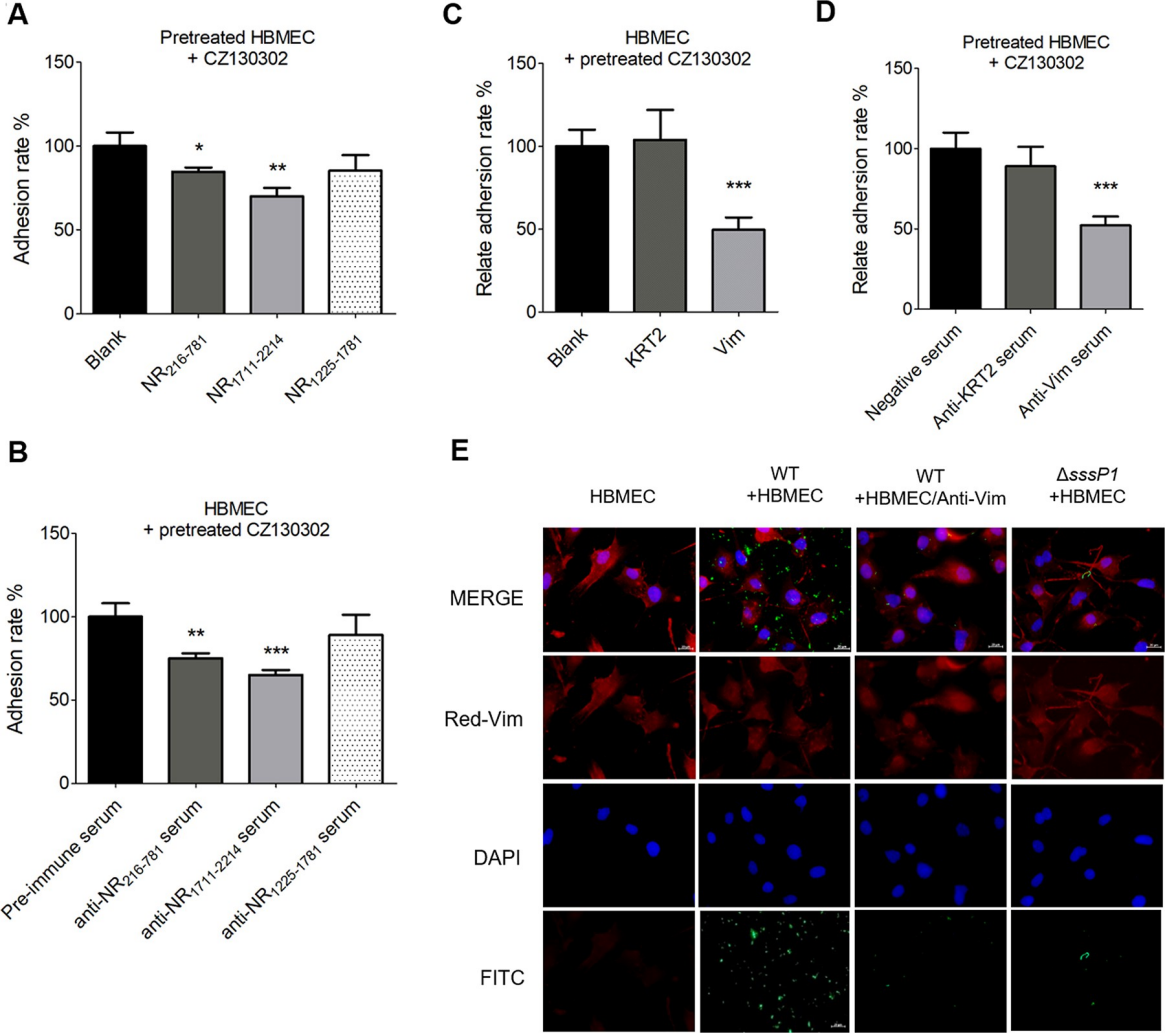
The adhesion of *S*. *suis* to HBMECs was attenuated by the blocking of vimentin, NR216-781 or NR1711-2214. All data are represented as mean ± SEM of triplicate samples (*** *P* < 0.001, ** *P* < 0.01, *P < 0.05). (A) The adhesion levels of *S*. *suis* strain CZ130302 were detected using the HBMECs pretreated with the NR_216-781_, NR_1711-2214_ and NR_1225-1781_ fragments. (B) The adhesion levels of *S*. *suis* strain CZ130302 to HBMECs were detected when anti-NR_216-781_ or anti-NR_1711-2214_ serums were added during co-incubation at MOI 100:1. The values were normalised against cells incubated with the pre-immune serum. (C) The adhesion levels of *S*. *suis* strain CZ130302 were detected using the HBMECs pretreated with the vimentin or Recombinant-keratin-2 (KRT2). KRT2 protein was use as the negative control. The cells without any treatments were used as the blank control. (D) The adhesion levels of *S*. *suis* strain CZ130302 to HBMECs were detected when the anti-vimentin or negative serums were added during the co-incubation at the MOI 100:1. (E) Confocal microscopy observation of vimentin skeleton and bacterial tracer. The images showed that the nuclei were stained with DAPI in blue, the vimentin was stained by IIF in red, and the *S*. *suis* cells with green fluorescent plasmid pKSM410-rss29 were observed in green.

The adhesion and adhesion inhibition assays were performed to verify the pathogenic roles of SssP1 amplified by the interaction with the vimentin of HBMECs. As shown in Fig 7A and 7B, the adhesion ability significantly attenuated when the CZ130302 cells were blocked by the antiserum of NR_216-781_ or NR_1711-2214_ (*P* < 0.01, *P* < 0.001), or the HBMECs were preincubated with the recombinant protein NR_216-781_ or NR_1711-2214_ (*P* < 0.05, *P* < 0.01), while this defect was not observed in the NR_1225-1781_ or anti-NR_1225-1781_ serum treated sample. Following assays were performed by blocking the vimentin of HBMECs using antiserum or blocking the SssP1 of *S*. *suis* by preincubating with vimentin. The results confirmed that the adhesion ability of the CZ130302 strain to HBMECs was significantly attenuated when the bacterial cells were preincubated with vimentin (Fig 7C) (*P* < 0.001). Similar attenuations were observed when HBMECs were blocked by the vimentin antibody (Fig 7D) (*P* < 0.001). Expectedly, the above attenuations were restored in the KRT2 treated strain CZ130302 or anti-KRT2 serum treated HBMECs to a similar level with the control sample. In addition, the indicated bacterial strains with the green fluorescent plasmid pKSM410-rss29 were observed using the fluorescence microscope to visualise the bacterial adhesion of *S*. *suis*. As shown in Fig 7E, the adhesion ability was significantly attenuated to a similar level of Δ*sssP1* when the vimentin antibody blocked the CZ130302. All the above results confirmed that the full virulence of *S*. *suis* to meningitis is significantly associated with the interaction between SssP1 and vimentin of HBMECs.

## Discussion

**Fig 8:**
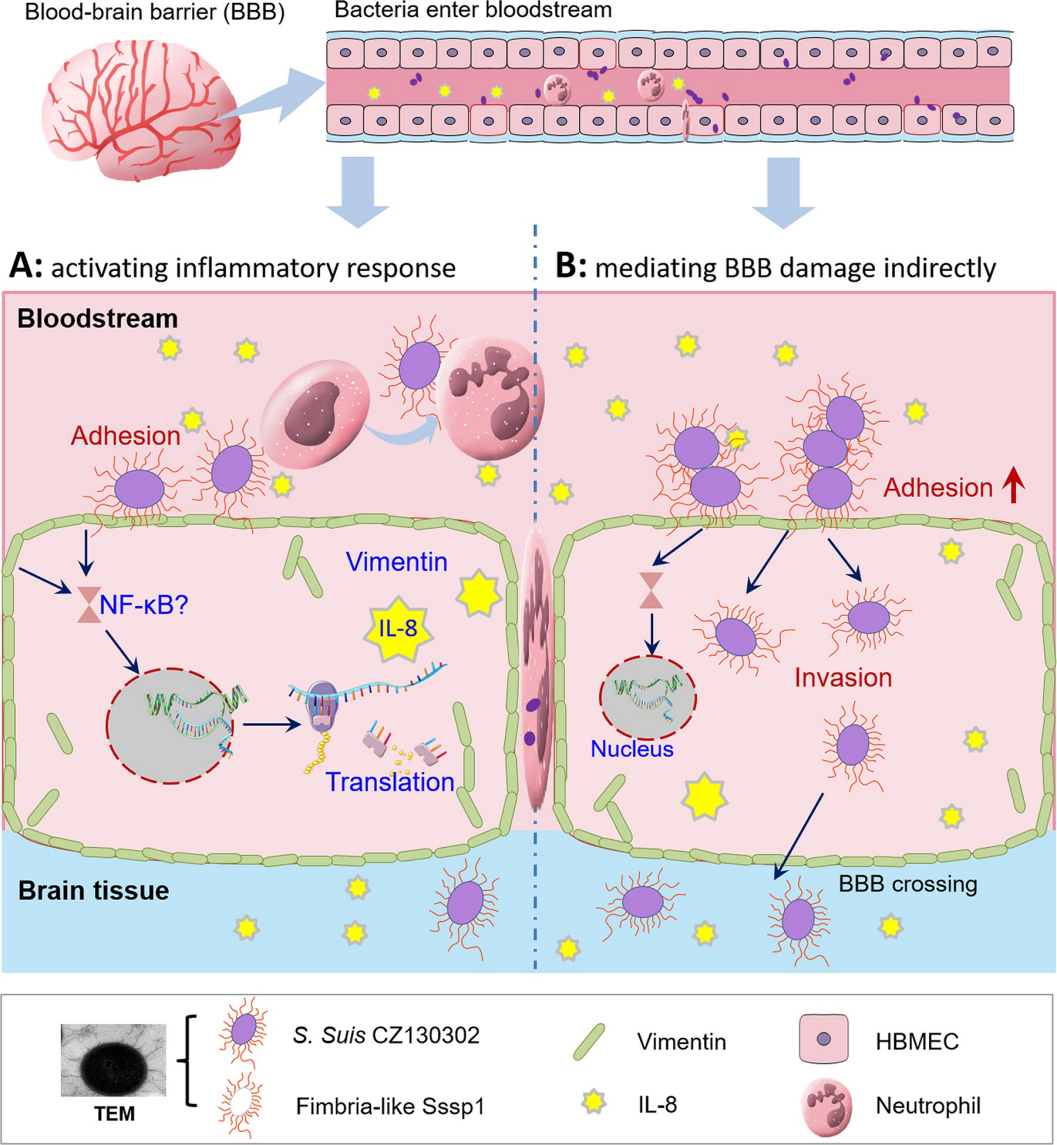
Summary of the role of the SssP1-vimentin interaction in promoting meningitis. SssP1 interacts with vimentin on the surface of HBMECs to promote *S*. *suis* attachment and induce robust inflammatory responses, which contributes to destroying the BBB to cause meningitis indirectly. The transmission electron microscope (TEM) image of *S*. *suis* strain CZ130302 showing the fimbria-like construction of SssP1 proteins was observed by this study, which was is consistent with a previous study of our lab [16].

Bacterial invasion and translocation across the BBB are critical steps during the development of meningitis, while the identities of specific factors necessary to facilitate *S*. *suis* infection have remained unclear. Here we report the discovery that both host vimentin and *S*. *suis* SssP1 are necessary for efficient penetration of the BBB and brain colonisation by *S*. *suis*. Furthermore, SssP1 drives a strong binding effect with the host vimentin, promoting the bacterial adhesion to and penetration of the BBB, and inducing a robust inflammatory response. This overall picture (Fig 8) of bacteria-host interaction markedly improves our understanding of host barrier penetration, and *S*. *suis* virulence during meningitis.

SssP1 is a 4,647 aa SRR glycoprotein exported by *S*. *suis* to assemble a fimbria-like component on the cell surface. Numerous studies have demonstrated that the SRR family proteins mediate the interaction with host cells using the nonrepeat (NR) domains, thus playing critical roles in bacterial pathogenicity [17,18]. Furthermore, the SRR proteins from different bacterial pathogens showed significant genetic variability, especially in the NR regions. Therefore, there is a great variety of substrates bind by the NR regions on host cells, such as keratin 4, keratin 10, and conjugated sialic acid [19,50–52]. In different *S*. *suis* strains, the SRR NR regions also showed significant variability, and the homologous NR region of SssP1 only encoded by some high-virulent *S*. *suis* isolates. In this study, vimentin was identified as the specific substrate bound by SssP1 on the surface of BBB model *in vitro*. Indeed, vimentin locates on the surface of numerous cells such as T cells, platelets, neutrophils, activated macrophages, vascular endothelial cells, and brain microvascular endothelial cells [21–25]. Thereinto, the T cells, platelets, neutrophils, and activated macrophages targeted by the SssP1-vimentin interaction may serve as inadvertent carriers of bacterial cells for the optimal proliferation in the bloodstream [20]. Relatively high pathogen concentrations in blood are considered a prerequisite for microorganisms to traverse the BBB [5], and the subsequent SssP1-vimentin interaction on vascular endothelial cells and brain microvascular endothelial cells may further facilitate pathogens penetrating this barrier. Vimentin on the surface has been reported to function as the primary receptor for bacterial adhesion or to activate the potential signalling pathways [53,54]. However, the vimentin also can be modified and secreted by macrophages [22] and endothelial cells [55], and its roles during microbial infections should warrant further studies.

To further explore the pathogenic roles of SssP1, we identified two fragments of the NR2 regions, NR_216-781_ and NR_1711-2214_, that are necessary for the interaction with vimentin during BBB penetration. SRR proteins have been reported to utilise their siglec-like (Ig-like) domains to bind platelet sialoglycans preferentially in diverse pathogens [20]. Our previous study has confirmed that the Ig-like domains of SssP1 showed binding capacities for human sialic acids and possessed apparent adherence to HBMECs [16]. In this study, two fragments, NR_1225-1781_ and NR_1711-2214_ contain an Ig-like domain, respectively, while only NR_1711-2214_ could interact with host vimentin and HBMECs, suggesting that the other domain of NR_1711-2214_, BLLF1 (herpes virus major outer envelope glycoprotein domain), might play vital roles for binding to vimentin. Otherwise, NR_216-781_ does not contain any known domains, and its interaction with vimentin or HBMECs could be abolished by the sialidase (Sigma) treatment, suggesting that NR_216-781_ bound to vimentin in a sialylation-dependent manner, while which was not mediated by the Ig-like domain. The homologous of SssP1 or other SRR proteins are widely encoded in the bacterial pathogens of *Streptococcal* species, and most of them could bind the surface sialoglycans of host cells. Suppose all these SRR proteins from diverse bacterial species have similar capacities to interact with the vimentin of BBB, which will significantly promote the exploration of the meningitis pathogenesis in a broader range of bacterial pathogens. Furthermore, a greater understanding of SssP1-vimentin interactions may prove applicable to other pathogens and provide significant insight and possible targets for developing novel therapeutics for meningitic infections. Our study has demonstrated that when the surface vimentin of HBMECs was blocked, the bacterial adhesion was significantly impaired, which suggests a new therapeutic approach to protect the CNS against bacterial invasion. Several recent studies have reported the strategy to block host-pathogen interaction to protect the brain from pathogen invasion during pneumococcal meningitis [56, 57]. Significantly, an increasing number of reports have demonstrated that vimentin is a central meningitic factor utilised by multiple bacterial pathogens to facilitate the crossing of the BBB and colonisation of the brain [35,38,53]. Therefore, early antibody blockade to vimentin may effectively resist the brain invasion of diverse bacterial pathogens, which provides a longer time window for proper choice of antibiotic, and thereby increase survival [57].

Except for the bacterial penetration of the BBB, paracellular migration of leukocyte and inflammatory activation occur at the BBB, which are the triad hallmark features of bacterial meningitis [54,58]. Paracellular migration of polymorphonuclear leukocytes across the BBB is not only crucial for host defence against pathogens, but it may also cause significant damage to the central nervous system tissues with a massive haemorrhage [59]. Furthermore, NF-κB signalling activation can significantly promote the central nervous system’s inflammation, damaging the brain from inflammatory injury during bacterial meningitis [60, 61]. Our study confirmed the roles of SssP1 in these three hallmark features *in vivo* by detecting the red blood cell count, white blood cell count, mRNA level of IL-8 encoding gene, bacterial load in CFS and brain, and pathological injury of the brain during meningitis caused by *S*. *suis* (Fig 8). Several studies have reported that vimentin plays a detrimental role in the host defence against meningitic infection by modulating the NF-κB signalling pathway to increase polymorphonuclear leukocytes recruitment and neuronal inflammation [35,38,53]. Therefore, the SssP1-vimentin interaction may facilitate brain damage from two perspectives, (i) traversing the BBB indirectly via enhancing bacterial adhesion and other unidentified pathways (ii) activating the inflammatory response within the central nervous system. Speculatively, these two perspectives may represent an important mechanism responsible for a coordinated modulation of both bacterial and host factors that contribute to the BBB penetration and bacterial diffusion within the central nervous system. Their cooperative contributions may represent a new paradigm in bacterial meningitis and lead to the development of novel strategies for preventing and treating *S*. *suis* infection.

Many meningitis-causing bacterial pathogens cross the BBB via the transcellular route by modifying or rearranging the actin cytoskeleton or via the paracellular route by disrupting the epithelial tight junction proteins (TJs) [41,42]. However, our study found that both the rearrangement of the actin cytoskeleton and disruption of TJs caused by the *S*. *suis* infection is independent of SssP1, which may be caused by: (i) SssP1 indeed does not perform the above biological functions, (ii) other virulence factors significantly facilitating the above phenotypes have obscured the actual effect of SssP1 in the above assays, and (iii) the roles of SssP1 are interrupted by several unknown reasons in these assays. These results suggest that the SssP1-vimentin interaction may contribute to traverse the BBB via indirect or unidentified pathways. Several previous studies have demonstrated that the specific interaction between IbeA and its primary receptor vimentin is necessary for the caveolae/lipid raft-dependent entry of *E*. *coli* K1 into HBMECs [38,53]. Otherwise, the IbeA-vimentin interaction mediates the polymorphonuclear leukocytes transmigration across the BBB [54], which may help bacterial pathogens hiding within the polymorphonuclear leukocytes to traverse the BBB via the infected Trojan horse mechanism. Whether similar mechanisms exist in Sssp1-vimentin interaction warrants further studies.

In summary, all the findings in this study indicate that the pathogenic features of *S*. *suis* meningitis are driven by specific interactions between the meningitic virulence factor SssP1 and its primary receptor vimentin (Fig 8). This ligand-receptor relationship provides a new angle to elucidate the pathogenesis of *S*. *suis*.

## Materials and methods

### Ethics statement

Five-week-old female specific pathogen free (SPF) BALB/c mice were purchased from Yangzhou University (Comparative Medicine Center). All animal experiments were performed in strict accordance with the animal welfare standards of the Animal Research Committee Guidelines of Jiangsu Province (License Number: SYXK (SU) 2017–0007), and approved by the Ethics Committee for Animal Experimentation of Nanjing Agricultural University.

### Bacterial and eukaryotic cell culture

The Chz serotype strain CZ130302 of *S*. *suis* was isolated from piglets with meningitis [15]. The Δ*sssP1* strain was constructed under the CZ130302 background by deleting the gene of *sssP1* [16]. All bacterial strains and plasmids used in this study are showed in Table 1. All strains of *S*. *suis* were cultured in Todd-Hewitt broth (THB, Becton-Dickinson) containing 3% fetal bovine serum, or solid medium containing 5% (*v/v*) sheep blood, in a 37°C incubator containing 5% CO_2_. In order to screen mutants, 100 μg/ml spectinomycin (Spc; Sigma-Aldrich) was added to THB medium. The *E*. *coli* strains were grown in Luria-Bertani (LB) medium. If necessary, 50 μg/ml kanamycin (Kan; Sigma-Aldrich) was added to the LB medium. HBMECs were preserved in our laboratory, while HA1800 cells were purchased from Wuhan aobei biotechnology co., LTD. Cells were cultured in Dulbecco’s modified Eagle medium (DMEM, Gibco) with 10% fetal bovine serum (FBS, Gibco) at 37°C in 5% CO_2_. The green fluorescent plasmid (pKSM410), generously donated by Kevin S. Mclver, was integrated with the strong promoter rss29 of *S*. *suis* to construct fluorescent tracer plasmid pKSM410-rss29.

### HBMECs adhesion and invasion assays

The HBMECs were cultured in 24-well cell plates (~5×10^5^ cells/well) and infected with treated bacterial suspension at a bacterium-to-cell ratio (multiplicity of infection, MOI) of 100:1. After that, the plates were immediately centrifuged at 800×*g* for 15 min and then incubated at 37°C for 2 h, followed with gently washed five times by sterile 1×PBS to remove unbound bacteria. Then, it was treated with 0.25% trypsin-EDTA (100 μL) for 10 min, and sterile deionised water (900 μL) was added to release bacteria. Then, the product was serially diluted ten-fold and dropped on the THA plate to count the bacterial colony and calculate the relative adhesion rate after the incubation at 37°C for 12 h. Quantitative invasion assays were performed similarly to the adhesion assay, except that the vancomycin and lysozyme treatment step for 1 h was added after the bacterial co-incubation with HBMECs. Each sample was evaluated in triplicate and repeated three times independently.

### Adhesion inhibition assays

Compared with the host cell adhesion assay, the procedure of co-incubation with the indicated inhibiting components was added into the adhesion inhibition assay. For antibody inhibition, the CZ130302 strain was co-incubated with anti-NR_216-781_ or anti-NR_1711-2214_ antibody at 37°C for 30 min, or the vimentin antibody blocked the HBMEC for 30 min. For protein binding inhibition, the CZ130302 strain was co-incubated with 100μg vimentin for 2 h at 37°C, or the HBMECs were incubated with 100μg NR_216-781_ or NR_1711-2214_ for 2 h at 37°C. The subsequent treatments were performed as the HBMECs adhesion assays.

### Determination of the IL-8 release level

In the logarithmic growth phase (OD_600_ = 0.6), the bacterial cells were collected and washed twice with PBS, and resuspended with DMEM culture medium to suitable concentrations. The HBMECs were cultured in 24-well cell plates until the cell fusion reached 80% (~5×10^5^ cells/well), infected with the treated bacterial suspension at the MOI 100:1, and centrifuged at 800×*g* for 10 min at room temperature. After incubation at 37°C in 5% CO_2_ atmosphere for 4 h, their supernatant was collected. Subsequently, the release levels of the IL-8 were determined using the Human IL-8 enzyme-linked immunosorbent assay (ELISA) kit (Bio-Techne) according to the manufacturer’s protocol.

### The permeability of BBB model *in vitro* during bacterial infection

Transwell chambers (1.12 cm^2^, 3μm) were coated with 10 μg/cm^2^ collagen type I (Sigma). The coating was dried overnight and then rinsed with DMEM. To construct the monolayer HBMECs BBB model, the 500 μL HBMECs (1×10^5^ cell/mL) were plated on the upper side of Transwell chambers with DMEM to balance the internal and external liquid level of the culture plates at 37°C with 5% CO_2_. For the monolayer HA1800 cells BBB model, the Transwells were placed upside down, and the 400 μL HA1800 cells (2×10^5^ cell/mL) were seeded on the bottom side of the Transwell chambers. Then, 8 h later, the Transwells were inverted and cultured with DMEM containing 10% FBS. To construct the joint HBMECs and HA1800 cells BBB model, the HA1800 cells were inversely inoculated and stationary cultured firstly. After the HA1800 cells reached a steady state, the HBMECs were inoculated on the upper side of Transwell chambers (S1 Fig). The TEER value was monitored, and the culture medium was replaced every two days until the value reached a stabilised threshold; then, the BBB models can be used for the study.

The log-phase bacterial cells of strains CZ130302 and Δ*sssP1* were collected and washed with PBS twice, and they were adjusted to a concentration of 2×10^5^ CFU/mL with DMEM without FBS. 500 μL diluted bacterial solution was then added into the upper chamber of the constructed BBB models and cultured at 37°C with 5% CO_2_. For the bacterial penetration test, the culture solutions from the bottom chambers of Transwell were collected in the indicated times and serially 10-fold diluted to plate on THA medium to enumerate CFU and calculate penetration rate. In addition, the sodium Fluorescein (FLU) was added to this infection model for the FLU penetration test and measured the fluorescence value (OD428) of the bottom chambers at the 2 h post-infection.

### TEER detection of BBB model

Trans-endothelial electrical resistance (TEER) was one of the most accurate and sensitive methods to detect the integrity of BBB *in vitro* [62]. After replacing the culture medium to remove the suspended cell debris, the TEER value was monitored for the constructed BBB models. The detection was performed in three different zones of the Transwell chamber, and the mean was calculated. The TEER value reached a stabilised threshold in about 4–6 days; then, the BBB models can be used for the study. The Transwell chamber without any cells was set as the blank control, and the final TEER value was calculated as the following formula: TEER/(Ω•cm_2_) = (RTotal-RBlank)×S.

### The permeability of BBB during bacterial infection *in vivo*

The permeability of BBB during *S*. *suis* infection was measured using the Evans Blue (EB) as the previous study reported [63]. Briefly, the challenged BALB/c mice were injected with 100 μl 2% EB via caudal vein at 12 h post-infection. Two hours later, the mice were anaesthetised and transcardially perfused with 0.9% NaCl containing EDTA to dilute the remaining EB in the blood vessels until the perfusate was clear. Then, the brain tissues were taken out and put into a 56°C oven. Two to three days later, the EB permeating into the brain was extracted by methanamide. Then, the Enzyme marker was used to measure the absorption value in 620 nm.

### Mouse infection assays and determination of bacterial load in viscera

Instead of the piglet infection model, the BALB/c mouse model is also widely used in the virulence assessment of SS2 [64,65]. Ten mice in each group were challenged with the indicated *S*. *suis* strain at a dose of 5 × 10^7^ CFU/mouse by intraperitoneal injection, and the clinical symptoms and death of mice were monitored every 12 h. The negative-control group was challenged with an equal volume of sterile PBS. Additionally, a bacterial load assay was conducted to evaluate the proliferation and invasion capacity of the indicated *S*. *suis* strains. Mice were injected with 5×10^7^ CFU/mouse, and their blood and brain were harvested, weighed, and homogenised in PBS at 6 h post-infection. Subsequently, the homogenates and blood were serially 10-fold diluted and plated on a THA medium to enumerate CFU.

### RNA isolation and RT-qPCR analysis

Briefly, total RNA from the infected host cells was extracted using the RNAiso Plus reagent (Takara), according to the manufacturer’s protocols, and residual genomic DNA was then removed by digestion with DNase I (TaKaRa). The PrimeScript RT reagent kit (TaKaRa) was used for cDNA synthesis instantly, and the RT-qPCR was performed using SYBR premix Ex Taq (TaKaRa) with gene-specific primers. The transcript of β-Actin encoding gene was served as the endogenous control to normalize the relative amount of target gene mRNA. The 2^−ΔΔCt^ method was used to calculate the relative transcript levels of the target gene.

### Histopathological section of the brain

The dying mice challenged with the indicated bacterial strains were euthanised, and the brain tissues were harvested and fixed in 4% paraformaldehyde. The samples were placed in 80%, 90%, 95% and 100% ethanol to dehydrate for 2 h. Then, the samples were embedded in paraffin, sliced, and pasted, and then stained with hematoxylin-eosin. Finally, it was observed under a microscope.

### Purification of recombinant protein and preparation of polyclonal antibody

The potential domains within SssP1-NR region were analyzed by the Web service of CD search (https://www.ncbi.nlm.nih.gov/Structure/cdd/wrpsb.cgi) and SWISS MODEL (https://swissmodel.expasy.org/) to guide the division of fragments. The nucleic acid sequences encoding indicated fragments of the SssP1-NR region were amplified (primers listed in S1 Table) with the genomic DNA of strain CZ130302. The PCR products were cloned into a pET-28a vector following the standard molecular cloning procedures. The recombinant fragments were purified by Ni-NTA Spin Columns (QIAGEN) from BL21 (DE3) carrying the recombinant pET-28a plasmid after IPTG induction (0.1 mM) for 6 h at 37°C. The New Zealand white rabbit was immunised using purified recombinant protein 100 μg with appropriate adjuvant via subcutaneous injection. The polyclonal antiserum was collected from immunised rabbit after ten days of the third immunisation.

### Pull-down assays

The specific method of His pull-down refers to Pierce His Protein interaction pull-down kit (Pierce Biotechnology, USA). The 600 μL original Ni beads homogenate was mixed with 1 mL precooled PBS and centrifuge at 2500 rpm in 4°C for 3 min. After discarding the supernatant, the beads were resuspended with 200 μL precooled PBS on the ice. The control group was only added 200 μL pretreated beads. The experimental groups were mixed 200 μL pretreated beads with 500 μg His protein, shakily incubated at 4°C for 60 min and washed by 1 mL PBST five times. Then, all groups were added into 1 mg cell total protein with shaking incubation overnight at 4°C. After thrice wash, 50 μL RIPA Buffer and 50 μL 2×Loading Buffer were added and boiled for 10 min. Samples were then centrifuged at 12000 rpm for 5 min, and the supernatants were collected for extraction of the proteolytic peptides using the specific solution (0.1% formic acid and 2% acetonitrile). The collected supernatant after a 10 min centrifugation at 13200 rpm in 4°C was transferred into the sample tube for mass spectrometry identification using the Thermo Scientific Q Exactive mass spectrometer.

### Far-Western blot and Western blot analyses

For Far-Western blot, vimentin or sialidase (Sigma) pretreated vimentin (for 0.5 h at 37°C) was separated by SDS-PAGE, then transferred to PVDF membranes (Bio-Rad) and blocked with 5% (w/v) skimmed milk for 2 h at 37°C. Subsequently, the membranes were washed thrice using TBST (TBS containing 0.01% Tween 20) and incubated with the recombinant fragments of SssP1 for 2 h at 37°C. After thrice wash, the processed membranes were incubated with the specific antibody (1:2000 dilution) (Thermo Fisher) for 1 h at 37°C. After another thrice wash, the membranes were stained with the HRP conjugated secondary antibodies (Thermo Fisher, 1:2000) for 1 h at 37°C. The positive bands were detected using the 3,3’-diaminobenzidine. Except for co-incubation with recombinant fragments of SssP1, the Western blot detecting ZO-1, occludin, claudin-5 and β-actin was performed in a similar procedure.

### Confocal Microscopy and indirect immunofluorescence analyses

Firstly, the fluorescent tracer plasmid pKSM410-rss29 was constructed as the description of S3 Fig to visualise the localisation of *S*. *suis*. Next, the HBMECs were cultivated with sterile glass coverslips in cell plates until the cell fusion reached 80%. Then, the strains of CZ130302 and Δ*sssP1* containing pKSM410-rss29 were respectively incubated 2 h with HBMECs. Next, the cells were fixed with 4% paraformaldehyde for 15 minutes and sealed with 1% BSA for 30 min. After that, cells were incubated with Alexa Fluor 555 phalloidin (Thermo Fisher) or other indicated antibodies for 1 h in the dark, and cells nuclei were stained by 4,6-diamidino-2-phenylindole (DAPI; KeyGEN BioTECH) for 5 min. Finally, the coverslips covered with cells were mounted on slides using 50% glycerinum and observed in the Leica Sp5 AOBS laser confocal layer by scanning.

An immunofluorescence microscopy assay was performed to visualise the localisation of SssP1 and vimentin during the interaction between *S*. *suis* and HBMECs. First, the HBMECs were seeded onto 24-well cell plates and treated with SssP1_NR216-781_ or SssP1_NR1711-2214_ (100 μg/well) for 1 h at 37°C. Then, cells were fixed with cold methanol at -20°C for 20 min and incubated with the appropriate antibody (1:1000 dilution) at 37°C for 1 hour. After thrice wash, the cells were incubated with the secondary antibody IgG-FITC or IgG-Cy3 (1:2000 dilution) for 30 minutes in the dark at 37°C. Around an hour later, the bacterial nucleus was stained by DAPI after washing three times. The treated samples were finally visualised on the laser scanning confocal microscopes (Carl Zeiss LSM710).

### Statistical analyses

All experiments were repeated at least three times. Statistical analyses were performed using the GraphPad Prism version 8.0 (GraphPad, LaJolla, CA, USA). The curves of bacterial loads in CFS, and the curves of TEER values in BBB models within the indicated times were analyzed with the log rank test. Data from *in vivo* colonization assays were analyzed by Mann–Whitney two-tailed U tests. Two-way *t*-test was performed for the qRT-PCR results, and one-way ANOVA was used for the other assays. For all tests, a *P* value < 0.05 were considered statistically significant (* *P* < 0.05, ** *P* < 0.01, *** *P* < 0.001, **** *P* < 0.0001), and all the data were shown as mean ± SEM.

## Supporting information

S1 Fig Construction of BBB models using the monolayer HBMECs, HA1800 or the joint HBMECs & HA1800 *in vitro*. (TIF)

S2 Fig SssP1 promotes *S*. *suis* to cross the HUVEC model *in vitro*. (A) The curves showed the TEER values within 4 days. (B) The curves showed the TEER values of the HUVEC model infected with the indicated *S*. *suis* strains. (C) The penetration rates of HUVEC model infected with the indicated strains were measured.
(TIF)

S3 Fig Construction of fluorescent plasmid pKSM410-rss29. The promoter region of rss29 from the genome of *S*. *suis* strain P1/7 was amplified by PCR, and inserted into the site cut by enzyme Stu1 in the plasmid pKSM410. The positive clone pKSM410-rss29 was identified and multiplicated in *E*. *coli* DH5α. Then the pKSM410-rss29 was transformed into the strains of CZ130302 and Δ*sssP1*. After identified by PCR, the positive clone strains of CZ130302 and Δ*sssP1* were observed by the Carl Zeiss LSM710 microscope.
(TIF)

S4 Fig The bacterial adhesion assays using the sialidase (Sigma) pretreated HBMECs. The adhesion levels of the indicated *S*. *suis* strains were detected at the MOI 100:1. The hyaluronidase (Sigma) pretreated cells was used as control.
(TIF)

S1 Table Bacterial strains, plasmids, and primers used in this study. (DOCX)

S2 Table Analysis of protein interaction between SssP1 and HBMEC cells. (DOCX)

S1 File Unedited western blots data in this study. (DOCX)
