# Supplementary figures and images for: SssP1, a Fimbria-like component of Streptococcus suis, binds to the vimentin of host cells and contributes to bacterial meningitis
Source: PLoS Pathog. 2022 Jul 19;18(7):e1010710. doi: 10.1371/journal.ppat.1010710 (PMC9337661; doi:10.1371/journal.ppat.1010710)

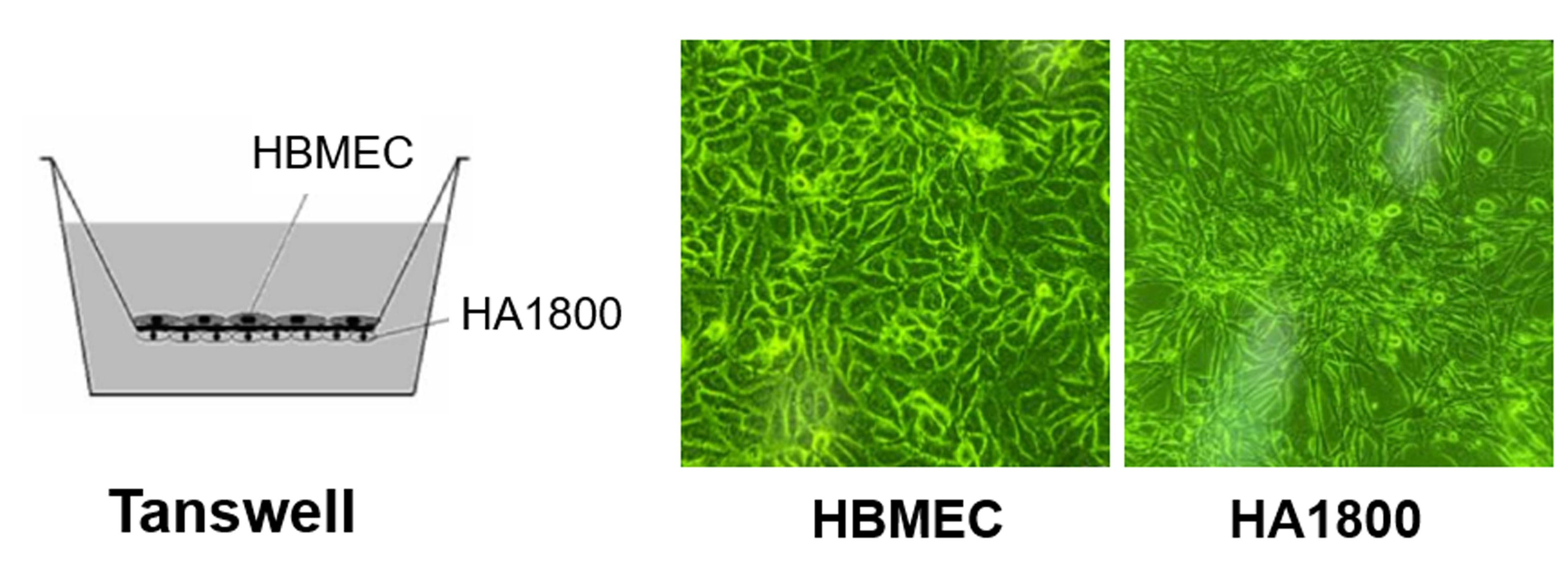

Supplement: S1 Fig — (TIF) [file ppat.1010710.s001.tif]

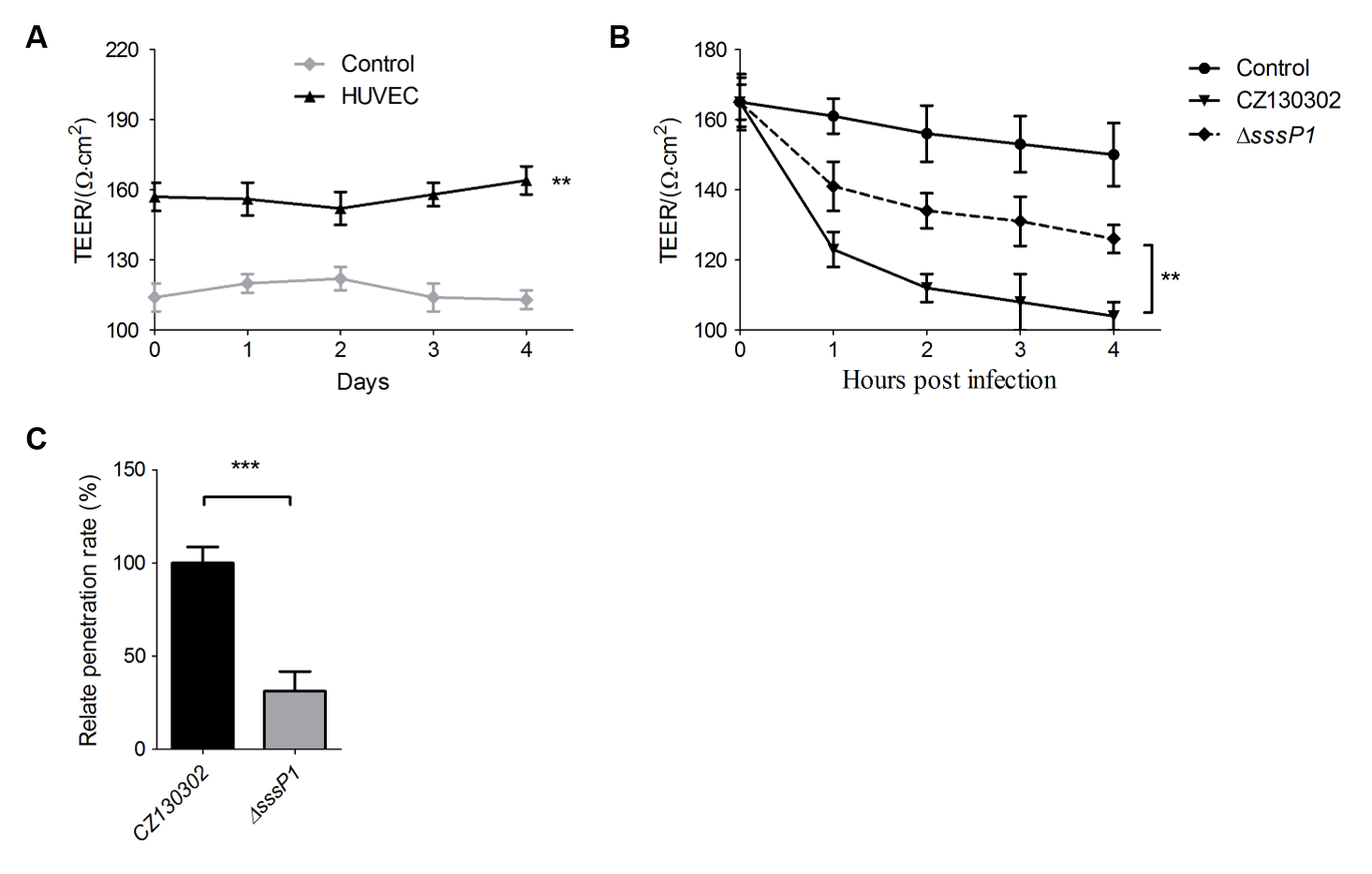

Supplement: S2 Fig — (A) The curves showed the TEER values within 4 days. (B) The curves showed the TEER values of the HUVEC model infected with the indicated S. suis strains. (C) The penetration rates of HUVEC model infected with the indicated strains were measured. (TIF) [file ppat.1010710.s002.tif]

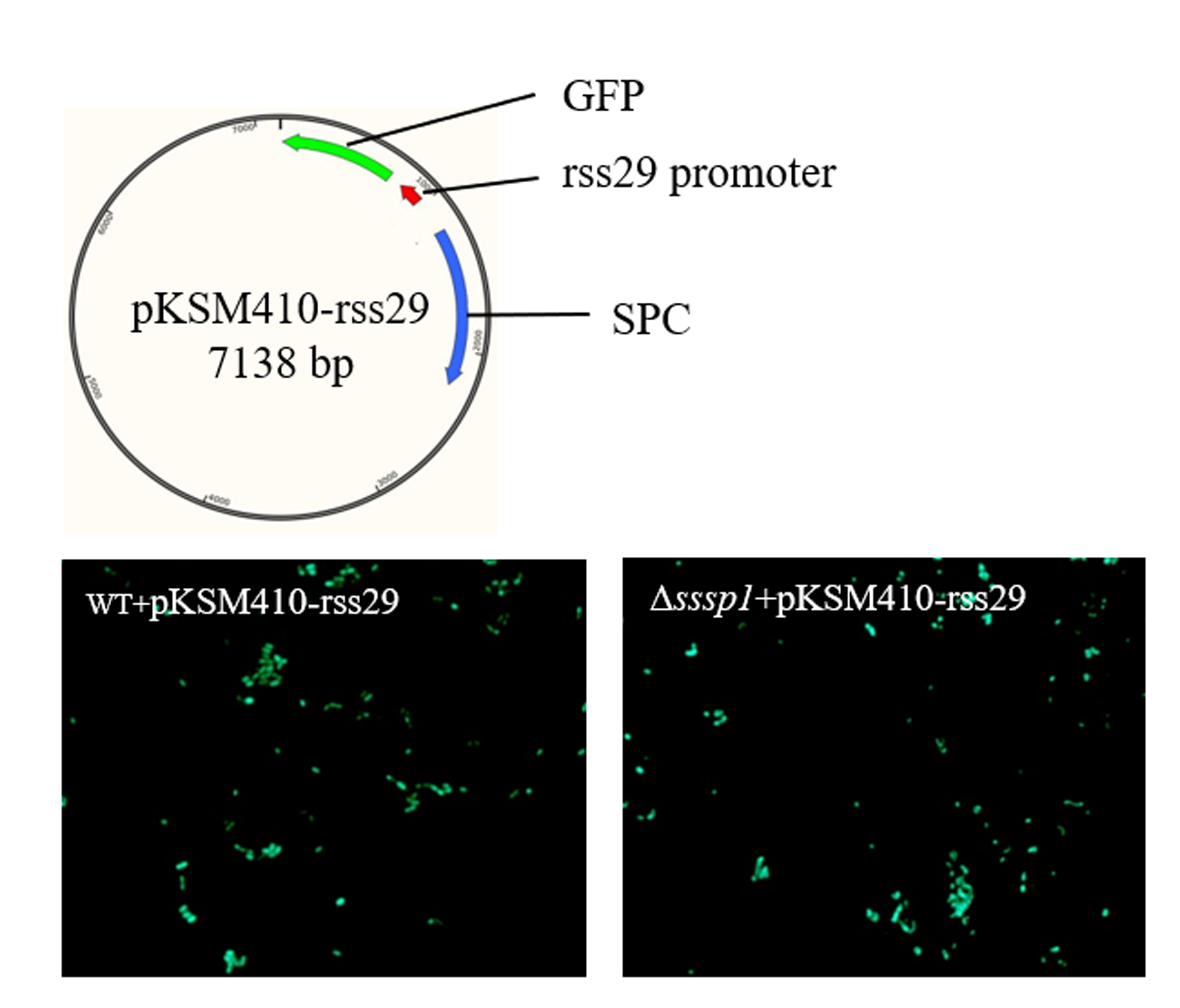

Supplement: S3 Fig — The promoter region of rss29 from the genome of S. suis strain P1/7 was amplified by PCR, and inserted into the site cut by enzyme Stu1 in the plasmid pKSM410. The positive clone pKSM410-rss29 was identified and multiplicated in E. coli DH5α. Then the pKSM410-rss29 was transformed into the strains of CZ130302 and ΔsssP1. After identified by PCR, the positive clone strains of CZ130302 and ΔsssP1 were observed by the Carl Zeiss LSM710 microscope. (TIF) [file ppat.1010710.s003.tif]

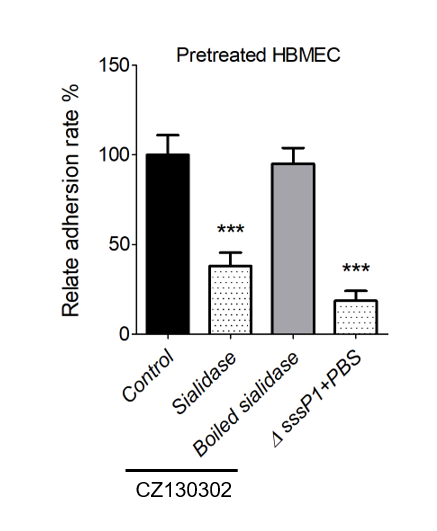

Supplement: S4 Fig — The adhesion levels of the indicated S. suis strains were detected at the MOI 100:1. The hyaluronidase (Sigma) pretreated cells was used as control. (TIF) [file ppat.1010710.s004.tif]

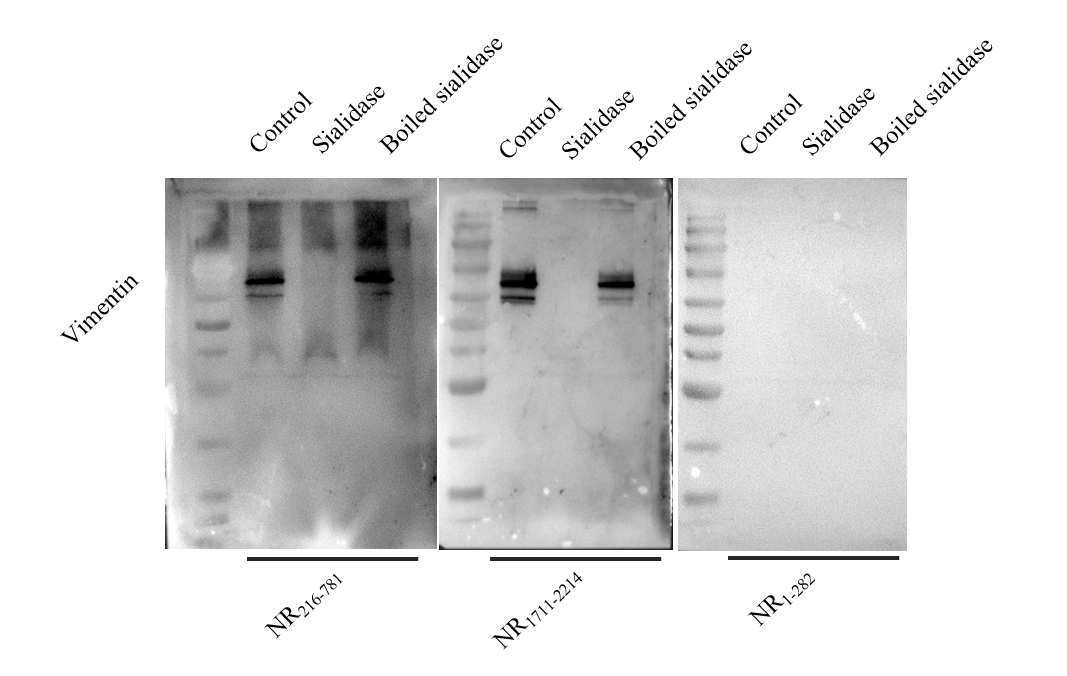


**For Fig. 6D**


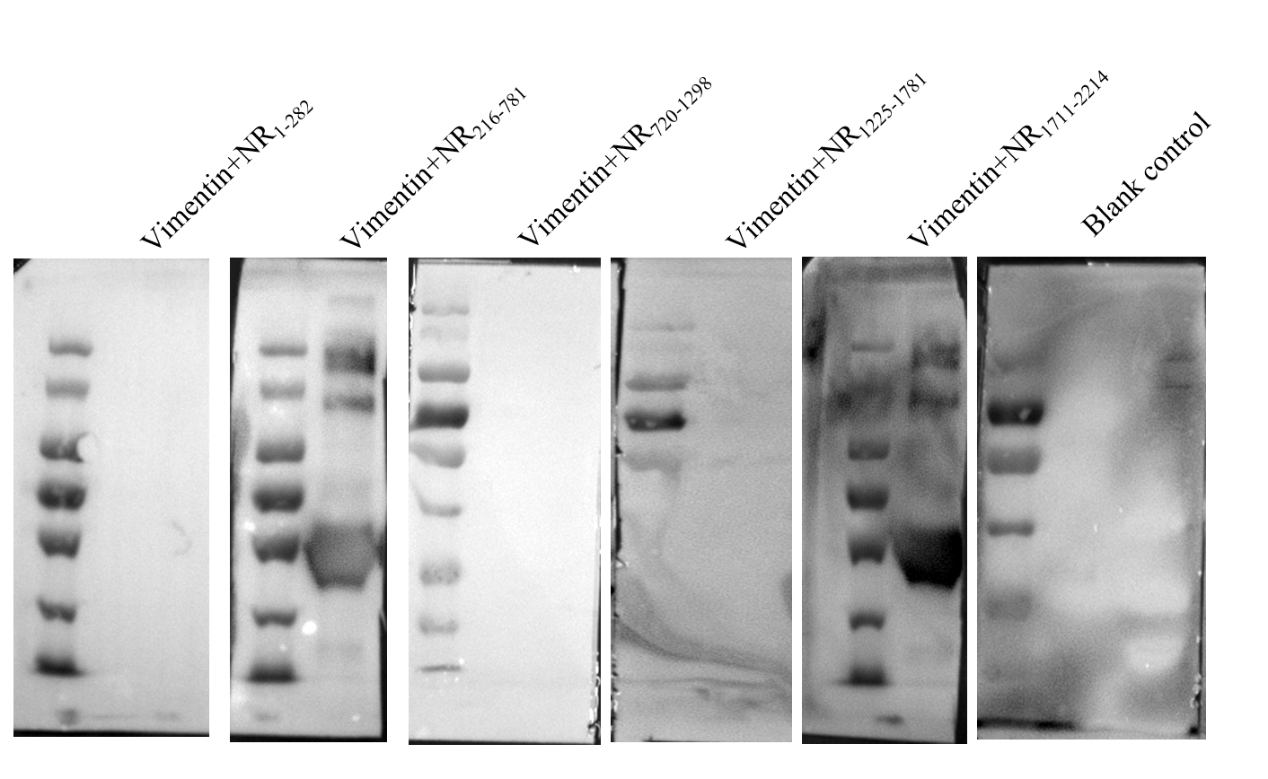


**For Fig. 6B**


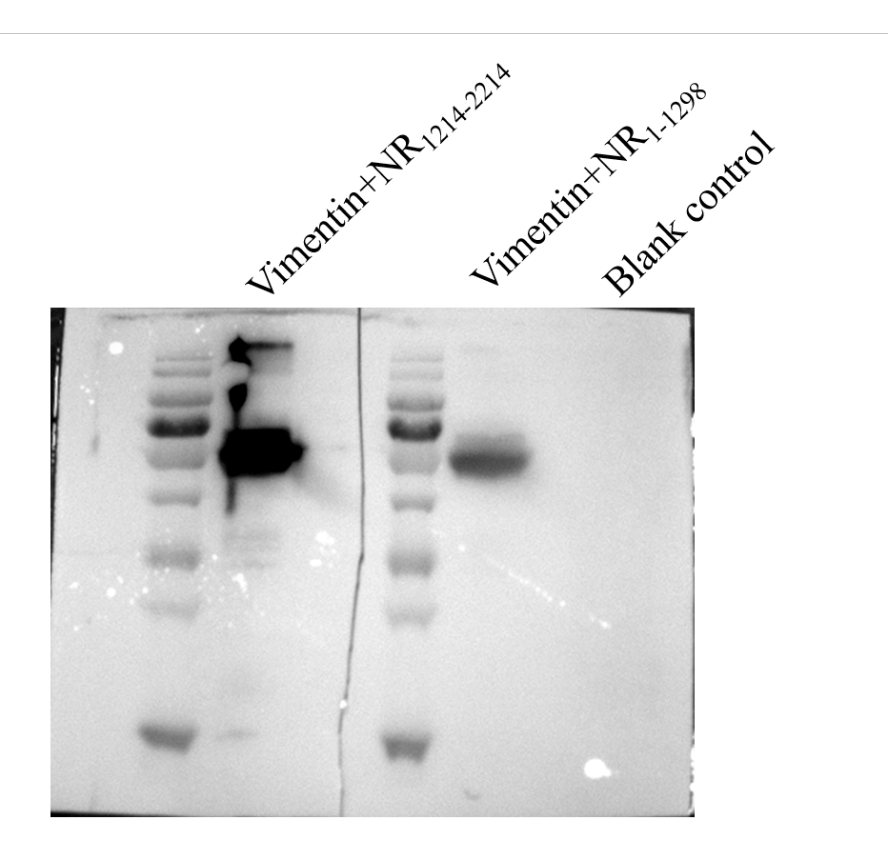


**For Fig. 5C**


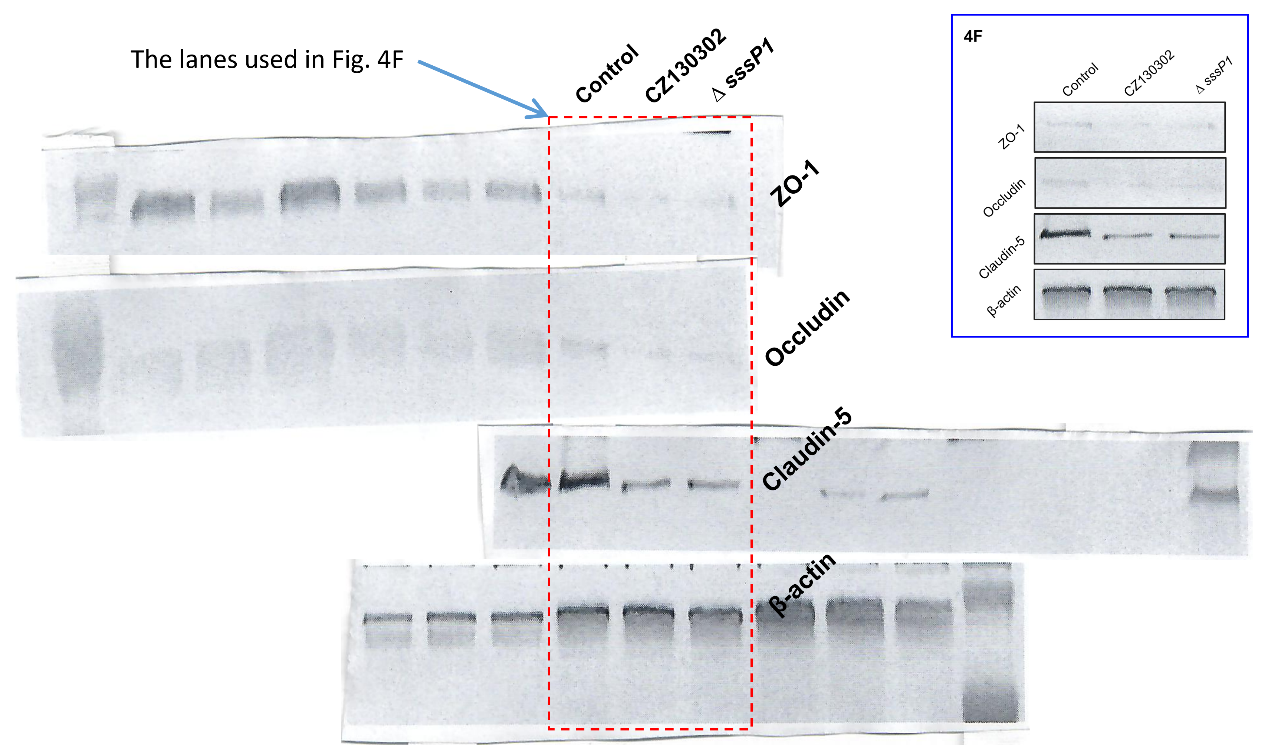


**For Fig. 4F**

(some other samples were from bEnd.3 cells)

Supplement: S1 File — (DOCX) [file ppat.1010710.s007.docx]
