# Supplementary material for: SssP1, a Fimbria-like component of Streptococcus suis, binds to the vimentin of host cells and contributes to bacterial meningitis
Source: PLoS Pathog. 2022 Jul 19;18(7):e1010710. doi: 10.1371/journal.ppat.1010710 (PMC9337661; doi:10.1371/journal.ppat.1010710)
Supplement: S1 Table — (DOCX) [file ppat.1010710.s005.docx]

**Table 1**

**Bacterial strains, plasmids, and primers used in this study.**

| **Strain, plasmid, or primer** | **Description^a^ or sequences (5′–3′)^b^** | **Sources or function** |
| --- | --- | --- |
| **Bacterial strains** |  |  |
| CZ130302  Δ*sssP1* | A novel variant serotype Chz of SS which caused acute meningitis in piglets  Deletion mutant of *sssP1* with CZ130302 background | Collected in our lab  Previous study |
| DH5α | Cloning host for maintaining the recombinant plasmids | Invitrogen |
| BL21(DE3) | Host for expressing the recombinant proteins | Invitrogen |
| **plasmids** |  |  |
| pET28a (+)  pET28a-SssP1NR_1-1298_  pET28a-SssP1_NR1225-2214_  pET28a-SssP1_NR1-282_  pET28a-SssP1_NR216-781_  pET28a-SssP1_NR720-1298_  pET28a-SssP1_NR1225-1781_  pET28a-SssP1_NR1711-2214_ | His-tag expressing vector, Kan^r^  pET-28a containing sssP1_NR1-1298_ gene, Kan^r^  pET-28a containing sssP1_NR1225-2214_ gene, Kan^r^  pET-28a containing sssP1_NR1-282_ gene, Kan^r^  pET-28a containing sssP1_NR216-781_ gene, Kan^r^  pET-28a containing sssP1 _NR720-1298_ gene, Kan^r^  pET-28a containing sssP1 _NR1225-1781_ gene, Kan^r^  pET-28a containing sssP1 _NR1711-2214_ gene, Kan^r^ | Invitrogen  This study  This study  This study  This study  This study  This study  This study |
| **Primers** |  |  |
| SssP1_NR1-1298_-L  SssP1_NR1-1298_-R  SssP1_NR1225-2214_-L  SssP1_NR1225-2214_-R  SssP1_NR1-282_-L  SssP1_NR1-282_-R  SssP1_NR216-781_-L  SssP1_NR216-781_-R  SssP1_NR720-1298_-L  SssP1_NR720-1298_-R  SssP1_NR1225-1781_-L  SssP1_NR1225-1781_-R  SssP1 _NR1711-2214_-L  SssP1 _NR1711-2214_-R  M-IL-8-F  M-IL-8-R  β-actin-F  β-actin-R | CgagctcATGACTGCAACTGAGTCTGGA  CCGctcgagAGCAGCTTGACCCTTTGGAGC  CgagctcACCAACGCAGCACCAGTTATT  CCGctcgagATTCCTGTCAGTAACCTTTAA  CgagctcATGACTGCAACTGAGTCTGGA  CCGctcgagATTAGTCGAGGATACATCATT  CgagctcCTAGGGTCTGTTGATGCTTCT  CCGctcgagGTAATCACTAGCATTCCCAAA  CgagctcGATACAGCCAATCCAGCTAAT  CCGctcgagAGCAGCTTGACCCTTTGGAGC  CgagctcACCAACGCAGCACCAGTTATT  CCGctcgagACCATTGCCAATTGCATCCAC  CgagctcAGTGATATCGAAGGGCTAACA  CCGctcgagATTCCTGTCAGTAACCTTTAA  AGGGCGGTCAAAAAGTTTGC  CAGGTACGATCCAGGCTTCC  CTTCCAGCCTTCCTTCCTGG  CTGTGTTGGCGTACAGGTCT | Primers for construction of SssP1NR1-1298  Primers for construction of SssP1 NR1225-2214  Primers for construction of SssP1NR1-282  Primers for construction of SssP1NR216-781  Primers for construction of  SssP1NR720-1298  Primers for construction of  SssP1NR1225-1781  Primers for construction of  SssP1 NR1711-2214  Primers for detecting the expression levels of the IL-8  Primers for detecting the expression levels of β-actin |

Kan^r^, kanamycin resistance cassette.
