# Supplementary material for: SssP1, a Fimbria-like component of Streptococcus suis, binds to the vimentin of host cells and contributes to bacterial meningitis
Source: PLoS Pathog. 2022 Jul 19;18(7):e1010710. doi: 10.1371/journal.ppat.1010710 (PMC9337661; doi:10.1371/journal.ppat.1010710)
Supplement: S2 Table — (DOCX) [file ppat.1010710.s006.docx]

**Table S2** Analysis of protein interaction between SssP1 and HBMEC cells.

The proteins binding both NR_1-1298_ and NR_1225-2214_ were showed with underlined font for further analysis in Fig. 5B. The proteins with top 7 scores corrected using the blank control were in bold font for further analysis in Table1.

| **Accession** | **Score** | **Mass** | **Matches** | **Sequences** | **emPAI** | **Localization** | **Protein description** |
| --- | --- | --- | --- | --- | --- | --- | --- |
| **Screening of receptor proteins interacting with protein SssP1-NR_1-1298_** | | | | | | | |
| **[sp\|P08670\|VIME_HUMAN](http://sale-depta-pc/mascot/cgi/master_results_2.pl?file=20180911%2FF002145.dat;pr.eh=4%2C4p;pr.page=4;pr.per_page=1;pr.show=proteins#tc:rf)** | **101** | **53676** | **14 (4)** | **10 (4)** | **0.27** | **Cytoplasm,**  **cytoskeleton,**  **nucleus,**  **synapse** | **Vimentin OS=Homo sapiens OX=9606 GN=VIM PE=1 SV=4** |
| [tr\|H6VRF8\|H6VRF8_HUMAN](http://sale-depta-pc/mascot/cgi/master_results_2.pl?file=20180911%2FF002145.dat;pr.eh=2%2C2p;pr.page=2;pr.per_page=1;pr.show=proteins#tc:rf) | 501 | 66184 | 45 (23) | 20 (15) | 1.39 | Cytoskeleton, mitochondrion | Keratin OS=Homo sapiens OX=9606 GN=KRT1 PE=3 SV=1 |
| [**sp\|P35908\|K22E_HUMAN**](http://sale-depta-pc/mascot/cgi/master_results_2.pl?file=20180911%2FF002145.dat;pr.eh=2%2C2p;pr.page=2;pr.per_page=1;pr.show=proteins#tc:rf) | **232** | **65678** | **19 (9)** | **11 (7)** | **0.41** | **Cytoplasm,**  **extracell** | **Keratin, type II cytoskeletal 2 epidermal OS=Homo sapiens OX=9606 GN=KRT2 PE=1 SV=2** |
| [**sp\|P13647\|K2C5_HUMAN**](http://sale-depta-pc/mascot/cgi/master_results_2.pl?file=20180911%2FF002145.dat;pr.eh=2%2C2p;pr.page=2;pr.per_page=1;pr.show=proteins#tc:rf) | **77** | **62568** | **9 (4)** | **7 (4)** | **0.23** | **Cytoskeleton,**  **mitochondrion,nucleus** | **Keratin, type II cytoskeletal 5 OS=Homo sapiens OX=9606 GN=KRT5 PE=1 SV=3** |
| [**sp\|P35527\|K1C9_HUMAN**](http://sale-depta-pc/mascot/cgi/master_results_2.pl?file=20180911%2FF002145.dat;pr.eh=3%2C3p;pr.page=3;pr.per_page=1;pr.show=proteins#tc:rf) | **362** | **62255** | **34 (15)** | **16 (10)** | **0.76** | **Extracell** | **Keratin, type I cytoskeletal 9 OS=Homo sapiens OX=9606 GN=KRT9 PE=1 SV=3** |
| [**sp\|P13645\|K1C10_HUMAN**](http://sale-depta-pc/mascot/cgi/master_results_2.pl?file=20180911%2FF002145.dat;pr.eh=3%2C3p;pr.page=3;pr.per_page=1;pr.show=proteins#tc:rf) | **347** | **59020** | **39 (15)** | **19 (10)** | **0.92** | **Extracell** | **Keratin, type I cytoskeletal 10 OS=Homo sapiens OX=9606 GN=KRT10 PE=1 SV=6** |
| [sp\|P02533\|K1C14_HUMAN](http://sale-depta-pc/mascot/cgi/master_results_2.pl?file=20180911%2FF002145.dat;pr.eh=3%2C3p;pr.page=3;pr.per_page=1;pr.show=proteins#tc:rf) | 132 | 51872 | 16 (7) | 7 (5) | 0.45 | Cytoplasm | Keratin, type I cytoskeletal 14 OS=Homo sapiens OX=9606 GN=KRT14 PE=1 SV=4 |
| [**tr\|Q1KLZ0\|Q1KLZ0_HUMAN**](http://sale-depta-pc/mascot/cgi/master_results_2.pl?file=20180911%2FF002145.dat;pr.eh=5%2C5p;pr.page=5;pr.per_page=1;pr.show=proteins#tc:rf) | **71** | **42052** | **10 (3)** | **7 (3)** | **0.25** | **Cytoskeleton** | **HCG15971, isoform CRA_a OS=Homo sapiens OX=9606 GN=PS1TP5BP1 PE=2 SV=1** |
| [**tr\|A0A075B6Z2\|A0A075B6Z2_HUMAN**](http://sale-depta-pc/mascot/cgi/master_results_2.pl?file=20180911%2FF002145.dat;pr.eh=6%2C6p;pr.page=6;pr.per_page=1;pr.show=proteins#tc:rf) | **48** | **2220** | **16 (6)** | **1 (1)** | **1.81** | **Extracell,** **plasma membrane** | **T cell receptor alpha joining 56 (Fragment) OS=Homo sapiens OX=9606 GN=****TRAJ56 PE=4 SV=1** |
| [tr\|A0A2P9AQT7\|A0A2P9AQT7_HUMAN](http://sale-depta-pc/mascot/cgi/master_results_2.pl?file=20180911%2FF002145.dat;pr.eh=7%2C7p;pr.page=7;pr.per_page=1;pr.show=proteins#tc:rf) | 21 | 25940 | 3 (1) | 2 (1) | 0.13 | Cytoplasm, nucleus | Phosphate-specific transport system accessory protein PhoU homolog OS=Homo sapiens OX=9606 GN=phoU PE=4 SV=1 |
| [tr\|B2R928\|B2R928_HUMAN](http://sale-depta-pc/mascot/cgi/master_results_2.pl?file=20180911%2FF002145.dat;pr.eh=8%2C8p;pr.page=8;pr.per_page=1;pr.show=proteins#tc:rf) | 20 | 96004 | 4 (1) | 1 (1) | 0.03 | Cytoplasm | Mitogen-activated protein kinase kinase kinase kinase OS=Homo sapiens OX=9606 PE=2 SV=1 |
| [tr\|H0YHX3\|H0YHX3_HUMAN](http://sale-depta-pc/mascot/cgi/master_results_2.pl?file=20180911%2FF002145.dat;pr.eh=9%2C9p;pr.page=9;pr.per_page=1;pr.show=proteins#tc:rf) | 20 | 16156 | 1 (1) | 1 (1) | 0.21 | Nucleus | Homeobox protein Hox-A4 (Fragment) OS=Homo sapiens OX=9606 GN=HOXA4 PE=3 SV=1 |
| [tr\|M0R043\|M0R043_HUMAN](http://sale-depta-pc/mascot/cgi/master_results_2.pl?file=20180911%2FF002145.dat;pr.eh=10%2C10p;pr.page=10;pr.per_page=1;pr.show=proteins#tc:rf) | 19 | 8174 | 4 (1) | 1 (1) | 0.43 | Nucleus | DNA replication complex GINS protein PSF2 (Fragment) OS=Homo sapiens OX=9606 GN=GINS2 PE=1 SV=1 |
| [tr\|Q5VT82\|Q5VT82_HUMAN](http://sale-depta-pc/mascot/cgi/master_results_2.pl?file=20180911%2FF002145.dat;pr.eh=11%2C11p;pr.page=11;pr.per_page=1;pr.show=proteins#tc:rf) | 19 | 114097 | 4 (1) | 1 (1) | 0.03 | Plasma membrane | Protocadherin-9 OS=Homo sapiens OX=9606 GN=PCDH9 PE=1 SV=1 |
| [tr\|A0A024R323\|A0A024R323_HUMAN](http://sale-depta-pc/mascot/cgi/master_results_2.pl?file=20180911%2FF002145.dat;pr.eh=12%2C12p;pr.page=12;pr.per_page=1;pr.show=proteins#tc:rf) | 16 | 38762 | 3 (1) | 1 (1) | 0.09 | Cytoplasm | Abhydrolase domain containing 6, isoform CRA_a OS=Homo sapiens OX=9606 GN=ABHD6 PE=4 SV=1 |
| **Screening of receptor proteins interacting with protein SssP1-NR_1225-2214_** | | | | | | | |
| [**sp\|P08670\|VIME_HUMAN**](http://sale-depta-pc/mascot/cgi/master_results_2.pl?file=20180911%2FF002145.dat;pr.eh=4%2C4p;pr.page=4;pr.per_page=1;pr.show=proteins#tc:rf) | **1583** | **53676** | **168 (94)** | **42 (37)** | **2.37** | **Cytoplasm,**  **cytoskeleton,**  **nucleus,**  **synapse** | **Vimentin OS=Homo sapiens OX=9606 GN=VIM PE=1 SV=4** |
| [sp\|P04264\|K2C1_HUMAN](http://sale-depta-pc/mascot/cgi/master_results_2.pl?file=20180911%2FF002144.dat;pr.eh=2%2C2p;pr.page=2;pr.per_page=1;pr.show=proteins#tc:rf) | 456 | 66170 | 46 (23) | 21 (13) | 1.17 | Mitochondrion | Keratin, type II cytoskeletal 1 OS=Homo sapiens OX=9606 GN=KRT1 PE=1 SV=6 |
| [**sp\|****P35908\|K22E_HUMAN**](http://sale-depta-pc/mascot/cgi/master_results_2.pl?file=20180911%2FF002144.dat;pr.eh=2%2C2p;pr.page=2;pr.per_page=1;pr.show=proteins#tc:rf) | **379** | **65678** | **42 (17)** | **22 (15)** | **1.08** | **Cytoplasm** | **Keratin, type II cytoskeletal 2 epidermal OS=Homo sapiens OX=9606 GN=KRT2 PE=1 SV=2** |
| [sp\|P08729\|K2C7_HUMAN](http://sale-depta-pc/mascot/cgi/master_results_2.pl?file=20180911%2FF002144.dat;pr.eh=2%2C2p;pr.page=2;pr.per_page=1;pr.show=proteins#tc:rf) | 360 | 51411 | 33 (15) | 17 (12) | 1.11 | Cytoskeleton,  Mitochondrion | Keratin, type II cytoskeletal 7 OS=Homo sapiens OX=9606 GN=KRT7 PE=1 SV=5 |
| [sp\|P05787\|K2C8_HUMAN](http://sale-depta-pc/mascot/cgi/master_results_2.pl?file=20180911%2FF002144.dat;pr.eh=2%2C2p;pr.page=2;pr.per_page=1;pr.show=proteins#tc:rf) | 174 | 53671 | 38 (13) | 24 (11) | 0.92 | Mitochondrion,Nucleus | Keratin, type II cytoskeletal 8 OS=Homo sapiens OX=9606 GN=KRT8 PE=1 SV=7 |
| [sp\|Q5XKE5\|K2C79_HUMAN](http://sale-depta-pc/mascot/cgi/master_results_2.pl?file=20180911%2FF002144.dat;pr.eh=2%2C2p;pr.page=2;pr.per_page=1;pr.show=proteins#tc:rf) | 97 | 58085 | 14 (3) | 8 (3) | 0.18 | Cytoskeleton,  Mitochondrion | Keratin, type II cytoskeletal 79 OS=Homo sapiens OX=9606 GN=KRT79 PE=1 SV=2 |
| [**sp\|****P13647\|K2C5_HUMAN**](http://sale-depta-pc/mascot/cgi/master_results_2.pl?file=20180911%2FF002144.dat;pr.eh=2%2C2p;pr.page=2;pr.per_page=1;pr.show=proteins#tc:rf) | **78** | **62568** | **18 (5)** | **11 (5)** | **0.29** | **Cytoskeleton,Mitochondrion, Nucleus** | **Keratin, type II cytoskeletal 5 OS=Homo sapiens OX=9606 GN=KRT5 PE=1 SV=3** |
| [sp\|Q15149\|PLEC_HUMAN](http://sale-depta-pc/mascot/cgi/master_results_2.pl?file=20180911%2FF002144.dat;pr.eh=3%2C3p;pr.page=3;pr.per_page=1;pr.show=proteins#tc:rf) | 883 | 533462 | 175 (59) | 114 (47) | 0.33 | Plasma  membrane, cytoskeleton | Plectin OS=Homo sapiens OX=9606 GN=PLEC PE=1 SV=3 |
| [**sp\|****P13645\|K1C10_HUMAN**](http://sale-depta-pc/mascot/cgi/master_results_2.pl?file=20180911%2FF002144.dat;pr.eh=4%2C4p;pr.page=4;pr.per_page=1;pr.show=proteins#tc:rf) | **619** | **59020** | **56 (33)** | **23 (17)** | **2.12** | **Cytoplasm, extracell** | **Keratin, type I cytoskeletal 10 OS=Homo sapiens OX=9606 GN=KRT10 PE=1 SV=6** |
| [sp\|P05783\|K1C18_HUMAN](http://sale-depta-pc/mascot/cgi/master_results_2.pl?file=20180911%2FF002144.dat;pr.eh=4%2C4p;pr.page=4;pr.per_page=1;pr.show=proteins#tc:rf) | 285 | 48029 | 37 (17) | 17 (10) | 1.08 | Mitochondrion, nucleus | Keratin, type I cytoskeletal 18 OS=Homo sapiens OX=9606 GN=KRT18 PE=1 SV=2 |
| [**tr\|****Q1KLZ0\|Q1KLZ0_HUMAN**](http://sale-depta-pc/mascot/cgi/master_results_2.pl?file=20180911%2FF002144.dat;pr.eh=5%2C5p;pr.page=5;pr.per_page=1;pr.show=proteins#tc:rf) | **555** | **42052** | **75 (33)** | **16 (13)** | **2.62** | **Cytoskeleton** | **HCG15971, isoform CRA_a OS=Homo sapiens OX=9606 GN=PS1TP5BP1 PE=2 SV=1** |
| [tr\|A0A024R1N1\|A0A024R1N1_HUMAN](http://sale-depta-pc/mascot/cgi/master_results_2.pl?file=20180911%2FF002144.dat;pr.eh=6%2C6p;pr.page=6;pr.per_page=1;pr.show=proteins#tc:rf) | 390 | 227646 | 91 (30) | 53 (21) | 0.43 | Plasma  Membrane, Cytoplasm, Nucleus | Myosin, heavy polypeptide 9, non-muscle, isoform CRA_a OS=Homo sapiens OX=9606 GN=MYH9 PE=3 SV=1 |
| [**sp\|****P35527\|K1C9_HUMAN**](http://sale-depta-pc/mascot/cgi/master_results_2.pl?file=20180911%2FF002144.dat;pr.eh=7%2C7p;pr.page=7;pr.per_page=1;pr.show=proteins#tc:rf) | **323** | **62255** | **31 (14)** | **14 (7)** | **0.59** | **Cytoplasm, extracell** | **Keratin, type I cytoskeletal 9 OS=Homo sapiens OX=9606 GN=KRT9 PE=1 SV=3** |
| [**tr\|****A0A075B6Z2\|A0A075B6Z2_HUMAN**](http://sale-depta-pc/mascot/cgi/master_results_2.pl?file=20180911%2FF002144.dat;pr.eh=8%2C8p;pr.page=8;pr.per_page=1;pr.show=proteins#tc:rf) | **96** | **2220** | **26 (11)** | **1 (1)** | **1.81** | **Extracell,** **plasma membrane** | **T cell receptor alpha joining 56 (Fragment) OS=Homo sapiens OX=9606 GN=TRAJ56 PE=4 SV=1** |
| [sp\|P16402\|H13_HUMAN](http://sale-depta-pc/mascot/cgi/master_results_2.pl?file=20180911%2FF002144.dat;pr.eh=9%2C9p;pr.page=9;pr.per_page=1;pr.show=proteins#tc:rf) | 84 | 22336 | 29 (3) | 7 (3) | 0.52 | Nucleus | Histone H1.3 OS=Homo sapiens OX=9606 GN=HIST1H1D PE=1 SV=2 |
| [tr\|Q0VAS5\|Q0VAS5_HUMAN](http://sale-depta-pc/mascot/cgi/master_results_2.pl?file=20180911%2FF002144.dat;pr.eh=10%2C10p;pr.page=10;pr.per_page=1;pr.show=proteins#tc:rf) | 66 | 11364 | 5 (3) | 4 (2) | 0.7 | Nucleus | Histone H4 OS=Homo sapiens OX=9606 GN=HIST1H4H PE=2 SV=1 |
| [tr\|A0A1W2PQM2\|A0A1W2PQM2_HUMAN](http://sale-depta-pc/mascot/cgi/master_results_2.pl?file=20180911%2FF002144.dat;pr.eh=11%2C11p;pr.page=11;pr.per_page=1;pr.show=proteins#tc:rf) | 47 | 37312 | 1 (1) | 1 (1) | 0.09 | Mitochondrion | Tubulin alpha-1C chain OS=Homo sapiens OX=9606 GN=TUBA1C PE=1 SV=1 |
| [tr\|W5X314\|W5X314_HUMAN](http://sale-depta-pc/mascot/cgi/master_results_2.pl?file=20180911%2FF002144.dat;pr.eh=12%2C12p;pr.page=12;pr.per_page=1;pr.show=proteins#tc:rf) | 43 | 10096 | 5 (1) | 4 (1) | 0.34 | Nucleus | Lamin A/C (Fragment) OS=Homo sapiens OX=9606 PE=3 SV=1 |
| [sp\|P02545\|LMNA_HUMAN](http://sale-depta-pc/mascot/cgi/master_results_2.pl?file=20180911%2FF002144.dat;pr.eh=12%2C12p;pr.page=12;pr.per_page=1;pr.show=proteins#tc:rf) | 41 | 74380 | 9 (2) | 8 (2) | 0.09 | Nucleus | Prelamin-A/C OS=Homo sapiens OX=9606 GN=LMNA PE=1 SV=1 |
| [tr\|A0A0C4DG40\|A0A0C4DG40_HUMAN](http://sale-depta-pc/mascot/cgi/master_results_2.pl?file=20180911%2FF002144.dat;pr.eh=13%2C13p;pr.page=13;pr.per_page=1;pr.show=proteins#tc:rf) | 42 | 1011226 | 23 (1) | 13 (1) |  | Plasma  membrane,  ,nucleus,  cytoplasm, | Nesprin-1 OS=Homo sapiens OX=9606 GN=SYNE1 PE=1 SV=1 |
| [tr\|A0A2R8Y5P9\|A0A2R8Y5P9_HUMAN](http://sale-depta-pc/mascot/cgi/master_results_2.pl?file=20180911%2FF002144.dat;pr.eh=14%2C14p;pr.page=14;pr.per_page=1;pr.show=proteins#tc:rf) | 41 | 209575 | 4 (2) | 2 (1) | 0.02 | Cytoplasm | Protein Shroom3 OS=Homo sapiens OX=9606 GN=SHROOM3 PE=4 SV=1 |
| [tr\|A0A024R2G9\|A0A024R2G9_HUMAN](http://sale-depta-pc/mascot/cgi/master_results_2.pl?file=20180911%2FF002144.dat;pr.eh=15%2C15p;pr.page=15;pr.per_page=1;pr.show=proteins#tc:rf) | 38 | 35329 | 3 (1) | 2 (1) | 0.09 | Endoplasmic reticulum | Oxidoreductase NAD-binding domain containing 1, isoform CRA_a OS=Homo sapiens OX=9606 GN=OXNAD1 PE=4 SV=1 |
| [tr\|A0A024QZH6\|A0A024QZH6_HUMAN](http://sale-depta-pc/mascot/cgi/master_results_2.pl?file=20180911%2FF002144.dat;pr.eh=16%2C16p;pr.page=16;pr.per_page=1;pr.show=proteins#tc:rf) | 32 | 139810 | 2 (1) | 1 (1) | 0.02 | Nucleus | Serine arginine-rich pre-mRNA splicing factor SR-A1, isoform CRA_a OS=Homo sapiens OX=9606 GN=SR-A1 PE=4 SV=1 |
| [tr\|L0R5A1\|L0R5A1_HUMAN](http://sale-depta-pc/mascot/cgi/master_results_2.pl?file=20180911%2FF002144.dat;pr.eh=17%2C17p;pr.page=17;pr.per_page=1;pr.show=proteins#tc:rf) | 32 | 11752 | 4 (1) | 1 (1) | 0.29 | Mitochondrion | Alternative protein CSF2RB OS=Homo sapiens OX=9606 GN=CSF2RB PE=4 SV=1 |
| [tr\|A0A2P9AN45\|A0A2P9AN45_HUMAN](http://sale-depta-pc/mascot/cgi/master_results_2.pl?file=20180911%2FF002144.dat;pr.eh=18%2C18p;pr.page=18;pr.per_page=1;pr.show=proteins#tc:rf) | 31 | 6464 | 6 (1) | 1 (1) | 0.56 | Extracell | Uncharacterized protein OS=Homo sapiens OX=9606 GN=BQ8482_30032 PE=4 SV=1 |
| [tr\|A0A2P9AQA1\|A0A2P9AQA1_HUMAN](http://sale-depta-pc/mascot/cgi/master_results_2.pl?file=20180911%2FF002144.dat;pr.eh=19%2C19p;pr.page=19;pr.per_page=1;pr.show=proteins#tc:rf) | 31 | 28641 | 3 (1) | 2 (1) | 0.12 | Plasma  membrane | Ion transport protein OS=Homo sapiens OX=9606 GN=BQ8482_340214 PE=4 SV=1 |
| [tr\|A0A2P9ACU3\|A0A2P9ACU3_HUMAN](http://sale-depta-pc/mascot/cgi/master_results_2.pl?file=20180911%2FF002144.dat;pr.eh=20%2C20p;pr.page=20;pr.per_page=1;pr.show=proteins#tc:rf) | 30 | 126794 | 10 (1) | 4 (1) | 0.03 | Mitochondrion | Sensor protein OS=Homo sapiens OX=9606 GN=BQ8482_110886 PE=4 SV=1 |
| [tr\|A0A087WUZ3\|A0A087WUZ3_HUMAN](http://sale-depta-pc/mascot/cgi/master_results_2.pl?file=20180911%2FF002144.dat;pr.eh=21%2C21p;pr.page=21;pr.per_page=1;pr.show=proteins#tc:rf) | 29 | 275457 | 17 (1) | 16 (1) | 0.01 | Plasma membrane, cytoplasm, cytoskeleton, nucleus | Spectrin beta chain OS=Homo sapiens OX=9606 GN=SPTBN1 PE=1 SV=1 |
| [tr\|B4DZY7\|B4DZY7_HUMAN](http://sale-depta-pc/mascot/cgi/master_results_2.pl?file=20180911%2FF002144.dat;pr.eh=22%2C22p;pr.page=22;pr.per_page=1;pr.show=proteins#tc:rf) | 29 | 61091 | 2 (1) | 1 (1) | 0.05 | Extracell | cDNA FLJ57022, highly similar to Growth-arrest-specific protein 6 OS=Homo sapiens OX=9606 PE=2 SV=1 |
| [sp\|Q5TCQ9\|MAGI3_HUMAN](http://sale-depta-pc/mascot/cgi/master_results_2.pl?file=20180911%2FF002144.dat;pr.eh=23%2C23p;pr.page=23;pr.per_page=1;pr.show=proteins#tc:rf) | 27 | 163761 | 2 (1) | 2 (1) | 0.02 | Nucleus | Membrane-associated guanylate kinase, WW and PDZ domain-containing protein 3 OS=Homo sapiens OX=9606 GN=MAGI3 PE=1 SV=3 |
| [tr\|A0A2P9AF85\|A0A2P9AF85_HUMAN](http://sale-depta-pc/mascot/cgi/master_results_2.pl?file=20180911%2FF002144.dat;pr.eh=24%2C24p;pr.page=24;pr.per_page=1;pr.show=proteins#tc:rf) | 27 | 39169 | 6 (1) | 3 (1) | 0.08 | Mitochondrion | D-amino acid oxidase OS=Homo sapiens OX=9606 GN=BQ8482_111723 PE=4 SV=1 |
| [tr\|A0A024R7U2\|A0A024R7U2_HUMAN](http://sale-depta-pc/mascot/cgi/master_results_2.pl?file=20180911%2FF002144.dat;pr.eh=26%2C26p;pr.page=26;pr.per_page=1;pr.show=proteins#tc:rf) | 26 | 33856 | 1 (1) | 1 (1) | 0.1 | Golgi apparatus | Membrane-associated ring finger (C3HC4) 8, isoform CRA_c OS=Homo sapiens OX=9606 GN=MARCH8 PE=4 SV=1 |
| [tr\|H0YAV3\|H0YAV3_HUMAN](http://sale-depta-pc/mascot/cgi/master_results_2.pl?file=20180911%2FF002144.dat;pr.eh=27%2C27p;pr.page=27;pr.per_page=1;pr.show=proteins#tc:rf) | 26 | 63231 | 2 (1) | 1 (1) | 0.05 | Nucleus | F-box only protein 11 (Fragment) OS=Homo sapiens OX=9606 GN=FBXO11 PE=1 SV=1 |
| [tr\|Q5VT37\|Q5VT37_HUMAN](http://sale-depta-pc/mascot/cgi/master_results_2.pl?file=20180911%2FF002144.dat;pr.eh=28%2C28p;pr.page=28;pr.per_page=1;pr.show=proteins#tc:rf) | 26 | 8360 | 4 (1) | 1 (1) | 0.42 | Nucleus | Zinc finger protein 669 OS=Homo sapiens OX=9606 GN=ZNF669 PE=4 SV=1 |
| [tr\|D3DSR2\|D3DSR2_HUMAN](http://sale-depta-pc/mascot/cgi/master_results_2.pl?file=20180911%2FF002144.dat;pr.eh=29%2C29p;pr.page=29;pr.per_page=1;pr.show=proteins#tc:rf) | 25 | 31916 | 1 (1) | 1 (1) | 0.1 | Nucleus | Polymerase (RNA) III (DNA directed) polypeptide D, 44kDa, isoform CRA_a OS=Homo sapiens OX=9606 GN=POLR3D PE=4 SV=1 |
| [tr\|A0A024R0R6\|A0A024R0R6_HUMAN](http://sale-depta-pc/mascot/cgi/master_results_2.pl?file=20180911%2FF002144.dat;pr.eh=30%2C30p;pr.page=30;pr.per_page=1;pr.show=proteins#tc:rf) | 25 | 141915 | 4 (1) | 3 (1) | 0.02 | Cytoplasm. nucleus | Symplekin, isoform CRA_a OS=Homo sapiens OX=9606 GN=SYMPK PE=4 SV=1 |
| [sp\|P36551\|HEM6_HUMAN](http://sale-depta-pc/mascot/cgi/master_results_2.pl?file=20180911%2FF002144.dat;pr.eh=31%2C31p;pr.page=31;pr.per_page=1;pr.show=proteins#tc:rf) | 24 | 50918 | 2 (1) | 2 (1) | 0.06 | Mitochondrion | Oxygen-dependent coproporphyrinogen-III oxidase, mitochondrial OS=Homo sapiens OX=9606 GN=CPOX PE=1 SV=3 |
| [tr\|H7C5D0\|H7C5D0_HUMAN](http://sale-depta-pc/mascot/cgi/master_results_2.pl?file=20180911%2FF002144.dat;pr.eh=32%2C32p;pr.page=32;pr.per_page=1;pr.show=proteins#tc:rf) | 24 | 15019 | 4 (1) | 1 (1) | 0.23 | Nucleus | Protein DENND6A (Fragment) OS=Homo sapiens OX=9606 GN=DENND6A PE=1 SV=1 |
| [tr\|A0A2P9AE75\|A0A2P9AE75_HUMAN](http://sale-depta-pc/mascot/cgi/master_results_2.pl?file=20180911%2FF002144.dat;pr.eh=33%2C33p;pr.page=33;pr.per_page=1;pr.show=proteins#tc:rf) | 24 | 23819 | 2 (1) | 1 (1) | 0.14 | Extracell, mitochondrion, nucleus | Uncharacterized protein OS=Homo sapiens OX=9606 GN=BQ8482_111346 PE=4 SV=1 |
| [sp\|P78527\|PRKDC_HUMAN](http://sale-depta-pc/mascot/cgi/master_results_2.pl?file=20180911%2FF002144.dat;pr.eh=34%2C34p;pr.page=34;pr.per_page=1;pr.show=proteins#tc:rf) | 24 | 473749 | 9 (1) | 6 (1) | 0.01 | Nucleus | DNA-dependent protein kinase catalytic subunit OS=Homo sapiens OX=9606 GN=PRKDC PE=1 SV=3 |
| [tr\|Q504W7\|Q504W7_HUMAN](http://sale-depta-pc/mascot/cgi/master_results_2.pl?file=20180911%2FF002144.dat;pr.eh=35%2C35p;pr.page=35;pr.per_page=1;pr.show=proteins#tc:rf) | 24 | 104312 | 4 (1) | 2 (1) | 0.03 | Nucleus | Oculocerebrorenal syndrome of Lowe OS=Homo sapiens OX=9606 GN=OCRL PE=2 SV=1 |
| [sp\|P0C0S5\|H2AZ_HUMAN](http://sale-depta-pc/mascot/cgi/master_results_2.pl?file=20180911%2FF002144.dat;pr.eh=36%2C36p;pr.page=36;pr.per_page=1;pr.show=proteins#tc:rf) | 23 | 13545 | 6 (1) | 3 (1) | 0.25 | Nucleus | Histone H2A.Z OS=Homo sapiens OX=9606 GN=H2AFZ PE=1 SV=2 |
| [tr\|K7EPB6\|K7EPB6_HUMAN](http://sale-depta-pc/mascot/cgi/master_results_2.pl?file=20180911%2FF002144.dat;pr.eh=37%2C37p;pr.page=37;pr.per_page=1;pr.show=proteins#tc:rf) | 23 | 4535 | 1 (1) | 1 (1) | 0.81 | Extracell, cytoplasm | KLRC4-KLRK1 readthrough (Fragment) OS=Homo sapiens OX=9606 GN=KLRC4-KLRK1 PE=4 SV=1 |
| [tr\|A0A2P9AHM2\|A0A2P9AHM2_HUMAN](http://sale-depta-pc/mascot/cgi/master_results_2.pl?file=20180911%2FF002144.dat;pr.eh=38%2C38p;pr.page=38;pr.per_page=1;pr.show=proteins#tc:rf) | 23 | 35138 | 1 (1) | 1 (1) | 0.09 | Plasma membrane, extracell | Sulfonate ABC transporter substrate-binding protein OS=Homo sapiens OX=9606 GN=BQ8482_160096 PE=4 SV=1 |
| [sp\|Q8NDA2\|HMCN2_HUMAN](http://sale-depta-pc/mascot/cgi/master_results_2.pl?file=20180911%2FF002144.dat;pr.eh=39%2C39p;pr.page=39;pr.per_page=1;pr.show=proteins#tc:rf) | 22 | 549223 | 5 (1) | 4 (1) | 0.01 | Extracell, plasma membrane,  lysosome, cytoplasm | Hemicentin-2 OS=Homo sapiens OX=9606 GN=HMCN2 PE=2 SV=3 |
| [tr\|F8WBY5\|F8WBY5_HUMAN](http://sale-depta-pc/mascot/cgi/master_results_2.pl?file=20180911%2FF002144.dat;pr.eh=40%2C40p;pr.page=40;pr.per_page=1;pr.show=proteins#tc:rf) | 21 | 4596 | 1 (1) | 1 (1) | 0.81 | Nucleus, extracell, cytoplasm | FOXL2 neighbor protein OS=Homo sapiens OX=9606 GN=FOXL2NB PE=4 SV=1 |
| [tr\|B7Z4I6\|B7Z4I6_HUMAN](http://sale-depta-pc/mascot/cgi/master_results_2.pl?file=20180911%2FF002144.dat;pr.eh=41%2C41p;pr.page=41;pr.per_page=1;pr.show=proteins#tc:rf) | 21 | 110961 | 2 (1) | 2 (1) | 0.03 | Nucleus | cDNA FLJ55581, highly similar to AF4/FMR2 family member 3 (Fragment) OS=Homo sapiens OX=9606 PE=2 SV=1 |
| [tr\|H0YHR8\|H0YHR8_HUMAN](http://sale-depta-pc/mascot/cgi/master_results_2.pl?file=20180911%2FF002144.dat;pr.eh=42%2C42p;pr.page=42;pr.per_page=1;pr.show=proteins#tc:rf) | 21 | 18227 | 1 (1) | 1 (1) | 0.19 | Cytoplasm | Cytosolic purine 5'-nucleotidase (Fragment) OS=Homo sapiens OX=9606 GN=NT5C2 PE=1 SV=1 |
| [tr\|B2RCB8\|B2RCB8_HUMAN](http://sale-depta-pc/mascot/cgi/master_results_2.pl?file=20180911%2FF002144.dat;pr.eh=43%2C43p;pr.page=43;pr.per_page=1;pr.show=proteins#tc:rf) | 20 | 129598 | 2 (1) | 2 (1) | 0.03 | Plasma membrane, cytoskeleton, extracell | cDNA, FLJ95971, highly similar to Homo sapiens protocadherin 12 (PCDH12), mRNA OS=Homo sapiens OX=9606 PE=2 SV=1 |
| [tr\|A0A024R1Y8\|A0A024R1Y8_HUMAN](http://sale-depta-pc/mascot/cgi/master_results_2.pl?file=20180911%2FF002144.dat;pr.eh=44%2C44p;pr.page=44;pr.per_page=1;pr.show=proteins#tc:rf) | 20 | 29127 | 1 (1) | 1 (1) | 0.11 | Extracell | Follistatin-like 3 (Secreted glycoprotein), isoform CRA_a OS=Homo sapiens OX=9606 GN=FSTL3 PE=4 SV=1 |
| [tr\|Q71RD1\|Q71RD1_HUMAN](http://sale-depta-pc/mascot/cgi/master_results_2.pl?file=20180911%2FF002144.dat;pr.eh=45%2C45p;pr.page=45;pr.per_page=1;pr.show=proteins#tc:rf) | 20 | 15813 | 3 (1) | 1 (1) | 0.22 | Extracell, mitochondrion | PP11647 OS=Homo sapiens OX=9606 PE=2 SV=1 |
| [sp\|Q8WVH0\|CPLX3_HUMAN](http://sale-depta-pc/mascot/cgi/master_results_2.pl?file=20180911%2FF002144.dat;pr.eh=46%2C46p;pr.page=46;pr.per_page=1;pr.show=proteins#tc:rf) | 19 | 17603 | 11 (1) | 1 (1) | 0.19 | Plasma membrane, nucleus, synapse | Complexin-3 OS=Homo sapiens OX=9606 GN=CPLX3 PE=2 SV=1 |
| [tr\|A0A2P9AFR2\|A0A2P9AFR2_HUMAN](http://sale-depta-pc/mascot/cgi/master_results_2.pl?file=20180911%2FF002144.dat;pr.eh=47%2C47p;pr.page=47;pr.per_page=1;pr.show=proteins#tc:rf) | 19 | 41886 | 1 (1) | 1 (1) | 0.08 | Extracell, nucleus | Uncharacterized protein OS=Homo sapiens OX=9606 GN=BQ8482_120004 PE=4 SV=1 |
| [tr\|A0A2P9AG02\|A0A2P9AG02_HUMAN](http://sale-depta-pc/mascot/cgi/master_results_2.pl?file=20180911%2FF002144.dat;pr.eh=48%2C48p;pr.page=48;pr.per_page=1;pr.show=proteins#tc:rf) | 19 | 20700 | 11 (1) | 3 (1) | 0.16 | Plasma membrane | Uncharacterized protein OS=Homo sapiens OX=9606 GN=BQ8482_120119 PE=4 SV=1 |
| [tr\|B3VL05\|B3VL05_HUMAN](http://sale-depta-pc/mascot/cgi/master_results_2.pl?file=20180911%2FF002144.dat;pr.eh=49%2C49p;pr.page=49;pr.per_page=1;pr.show=proteins#tc:rf) | 18 | 11536 | 4 (1) | 2 (1) | 0.3 | Extracell,  mitochondrion | Beta globin (Fragment) OS=Homo sapiens OX=9606 PE=3 SV=1 |
| [tr\|Q8IWY7\|Q8IWY7_HUMAN](http://sale-depta-pc/mascot/cgi/master_results_2.pl?file=20180911%2FF002144.dat;pr.eh=50%2C50p;pr.page=50;pr.per_page=1;pr.show=proteins#tc:rf) | 18 | 183893 | 9 (1) | 5 (1) | 0.02 | Cytoplasm,  nucleus | Tau-tubulin kinase 2 OS=Homo sapiens OX=9606 GN=TTBK2 PE=1 SV=1 |
| [tr\|B4DFB5\|B4DFB5_HUMAN](http://sale-depta-pc/mascot/cgi/master_results_2.pl?file=20180911%2FF002144.dat;pr.eh=51%2C51p;pr.page=51;pr.per_page=1;pr.show=proteins#tc:rf) | 17 | 11548 | 2 (1) | 1 (1) | 0.3 | Cytoplasm | cDNA FLJ60467, moderately similar to Aminoacylase-1 (EC 3.5.1.14) OS=Homo sapiens OX=9606 PE=2 SV=1 |
| [tr\|A0A024R7U5\|A0A024R7U5_HUMAN](http://sale-depta-pc/mascot/cgi/master_results_2.pl?file=20180911%2FF002144.dat;pr.eh=52%2C52p;pr.page=52;pr.per_page=1;pr.show=proteins#tc:rf) | 17 | 24757 | 3 (1) | 2 (1) | 0.14 | Cytoplasm, endoplasmic reticulum | EF-hand calcium binding domain 1, isoform CRA_b OS=Homo sapiens OX=9606 GN=EFCAB1 PE=4 SV=1 |
| [tr\|H3BR13\|H3BR13_HUMAN](http://sale-depta-pc/mascot/cgi/master_results_2.pl?file=20180911%2FF002144.dat;pr.eh=53%2C53p;pr.page=53;pr.per_page=1;pr.show=proteins#tc:rf) | 17 | 18665 | 1 (1) | 1 (1) | 0.18 | Nucleus | RNA exonuclease 5 (Fragment) OS=Homo sapiens OX=9606 GN=REXO5 PE=1 SV=8 |
| [tr\|H7BZT5\|H7BZT5_HUMAN](http://sale-depta-pc/mascot/cgi/master_results_2.pl?file=20180911%2FF002144.dat;pr.eh=54%2C54p;pr.page=54;pr.per_page=1;pr.show=proteins#tc:rf) | 17 | 58662 | 1 (1) | 1 (1) | 0.06 | Nucleus | Zinc finger protein 185 OS=Homo sapiens OX=9606 GN=ZNF185 PE=1 SV=2 |
| [tr\|A8MTF8\|A8MTF8_HUMAN](http://sale-depta-pc/mascot/cgi/master_results_2.pl?file=20180911%2FF002144.dat;pr.eh=55%2C55p;pr.page=55;pr.per_page=1;pr.show=proteins#tc:rf) | 17 | 28606 | 5 (1) | 3 (1) | 0.12 | Endoplasmic reticulum, extracell | Protein FAM3B OS=Homo sapiens OX=9606 GN=FAM3B PE=1 SV=2 |
| [sp\|Q8WXH0\|SYNE2_HUMAN](http://sale-depta-pc/mascot/cgi/master_results_2.pl?file=20180911%2FF002144.dat;pr.eh=56%2C56p;pr.page=56;pr.per_page=1;pr.show=proteins#tc:rf) | 16 | 801817 | 18 (1) | 13 (1) |  | Cytoskeleton | Nesprin-2 OS=Homo sapiens OX=9606 GN=SYNE2 PE=1 SV=3 |
| [sp\|O60673\|REV3L_HUMAN](http://sale-depta-pc/mascot/cgi/master_results_2.pl?file=20180911%2FF002144.dat;pr.eh=57%2C57p;pr.page=57;pr.per_page=1;pr.show=proteins#tc:rf) | 16 | 355976 | 17 (1) | 9 (1) | 0.01 | Nucleus | DNA polymerase zeta catalytic subunit OS=Homo sapiens OX=9606 GN=REV3L PE=1 SV=2 |
| [tr\|A0A2R8Y880\|A0A2R8Y880_HUMAN](http://sale-depta-pc/mascot/cgi/master_results_2.pl?file=20180911%2FF002144.dat;pr.eh=58%2C58p;pr.page=58;pr.per_page=1;pr.show=proteins#tc:rf) | 16 | 198157 | 3 (1) | 3 (1) | 0.02 | Nucleus | E3 ubiquitin-protein ligase RBBP6 (Fragment) OS=Homo sapiens OX=9606 GN=RBBP6 PE=4 SV=1 |
| [tr\|Q8NAC4\|Q8NAC4_HUMAN](http://sale-depta-pc/mascot/cgi/master_results_2.pl?file=20180911%2FF002144.dat;pr.eh=59%2C59p;pr.page=59;pr.per_page=1;pr.show=proteins#tc:rf) | 16 | 74043 | 1 (1) | 1 (1) | 0.04 | Nucleus | cDNA FLJ35587 fis, clone SPLEN2007350 OS=Homo sapiens OX=9606 PE=2 SV=1 |
| [tr\|E9PBZ0\|E9PBZ0_HUMAN](http://sale-depta-pc/mascot/cgi/master_results_2.pl?file=20180911%2FF002144.dat;pr.eh=60%2C60p;pr.page=60;pr.per_page=1;pr.show=proteins#tc:rf) | 16 | 8958 | 1 (1) | 1 (1) | 0.39 | Cytoplasm | Bis(5'-adenosyl)-triphosphatase (Fragment) OS=Homo sapiens OX=9606 GN=FHIT PE=1 SV=8 |
| [sp\|Q2WGJ6\|KLH38_HUMAN](http://sale-depta-pc/mascot/cgi/master_results_2.pl?file=20180911%2FF002144.dat;pr.eh=61%2C61p;pr.page=61;pr.per_page=1;pr.show=proteins#tc:rf) | 16 | 66524 | 3 (1) | 2 (1) | 0.05 | Cytoskeleton | Kelch-like protein 38 OS=Homo sapiens OX=9606 GN=KLHL38 PE=1 SV=3 |
| [tr\|H3BRK2\|H3BRK2_HUMAN](http://sale-depta-pc/mascot/cgi/master_results_2.pl?file=20180911%2FF002144.dat;pr.eh=62%2C62p;pr.page=62;pr.per_page=1;pr.show=proteins#tc:rf) | 15 | 4736 | 1 (1) | 1 (1) | 0.77 | Mitochondrion, extracell, cytoplasm, nucleus | Transcription factor 12 (Fragment) OS=Homo sapiens OX=9606 GN=TCF12 PE=1 SV=1 |
| **Blank control group** | | | | | | | |
| [K2C1_HUMAN](http://sale-depta-pc/mascot/cgi/master_results_2.pl?file=20180911%2FF002147.dat;pr.eh=1%2C1p;pr.page=1;pr.per_page=1;pr.show=proteins#tc:rf) | 322 | 66170 | 24 (12) | 16 (11) | 0.7 | Mitochondrion | Keratin, type II cytoskeletal 1 OS=Homo sapiens OX=9606 GN=KRT1 PE=1 SV=6 |
| [K22E_HUMAN](http://sale-depta-pc/mascot/cgi/master_results_2.pl?file=20180911%2FF002147.dat;pr.eh=1%2C1p;pr.page=1;pr.per_page=1;pr.show=proteins#tc:rf) | 125 | 65678 | 9 (4) | 7 (4) | 0.22 | Cytoplasm | Keratin, type II cytoskeletal 2 epidermal OS=Homo sapiens OX=9606 GN=KRT2 PE=1 SV=2 |
| [K1C10_HUMAN](http://sale-depta-pc/mascot/cgi/master_results_2.pl?file=20180911%2FF002147.dat;pr.eh=2%2C2p;pr.page=2;pr.per_page=1;pr.show=proteins#tc:rf) | 234 | 59020 | 16 (11) | 10 (8) | 0.72 | Cytoplasm, extracell | Keratin, type I cytoskeletal 10 OS=Homo sapiens OX=9606 GN=KRT10 PE=1 SV=6 |
| [K1C9_HUMAN](http://sale-depta-pc/mascot/cgi/master_results_2.pl?file=20180911%2FF002147.dat;pr.eh=2%2C2p;pr.page=2;pr.per_page=1;pr.show=proteins#tc:rf) | 181 | 62255 | 13 (8) | 8 (5) | 0.36 | Cytoplasm, extracell | Keratin, type I cytoskeletal 9 OS=Homo sapiens OX=9606 GN=KRT9 PE=1 SV=3 |
| [A0A075B6Z2_HUMAN](http://sale-depta-pc/mascot/cgi/master_results_2.pl?file=20180911%2FF002147.dat;pr.eh=3%2C3p;pr.page=3;pr.per_page=1;pr.show=proteins#tc:rf) | 33 | 2220 | 9 (3) | 1 (1) | 1.81 | Extracell, plasma membrane | T cell receptor alpha joining 56 (Fragment) OS=Homo sapiens OX=9606 GN=TRAJ56 PE=4 SV=1 |
| [B4DXP8_HUMAN](http://sale-depta-pc/mascot/cgi/master_results_2.pl?file=20180911%2FF002147.dat;pr.eh=4%2C4p;pr.page=4;pr.per_page=1;pr.show=proteins#tc:rf) | 27 | 65444 | 1 (1) | 1 (1) | 0.05 | Cytoplasm | Kinesin-like protein OS=Homo sapiens OX=9606 PE=2 SV=1 |
| [Q6ZS31_HUMAN](http://sale-depta-pc/mascot/cgi/master_results_2.pl?file=20180911%2FF002147.dat;pr.eh=5%2C5p;pr.page=5;pr.per_page=1;pr.show=proteins#tc:rf) | 26 | 19500 | 3 (1) | 1 (1) | 0.17 | Extracell | cDNA FLJ45872 fis, clone OCBBF3005843 OS=Homo sapiens OX=9606 PE=2 SV=1 |
| [A0A2R8Y793_HUMAN](http://sale-depta-pc/mascot/cgi/master_results_2.pl?file=20180911%2FF002147.dat;pr.eh=6%2C6p;pr.page=6;pr.per_page=1;pr.show=proteins#tc:rf) | 21 | 34405 | 4 (1) | 3 (1) | 0.1 | Cytoskeleton | Actin, cytoplasmic 1 (Fragment) OS=Homo sapiens OX=9606 GN=ACTB PE=4 SV=1 |
